# The inferior vena cava: anatomical variants and acquired pathologies

**DOI:** 10.1186/s13244-021-01066-7

**Published:** 2021-08-30

**Authors:** Simon J. Li, Jean Lee, Jonathan Hall, Tom R. Sutherland

**Affiliations:** 1 Medical Imaging Department, St Vincent’s Hospital Melbourne, 41 Victoria Parade, Fitzroy, VIC 3065 Australia; 2 Department of Radiology, Austin Health, Heidelberg, VIC Australia; 3 Faculty of Medicine, Dentistry and Health Sciences, University of Melbourne, Parkville, VIC Australia

**Keywords:** Anatomical variations, Embryology, Inferior vena cava (IVC), Thrombus, Tumour

## Abstract

The inferior vena cava (IVC) is the largest vein in the body, draining blood from the abdomen, pelvis and lower extremities. This pictorial review summarises normal anatomy and embryological development of the IVC. In addition, we highlight a wide range of anatomical variants, acquired pathologies and a common pitfall in imaging of the IVC. This information is essential for clinical decision making and to reduce misdiagnosis.

## Teaching points

- IVC anomalies are the result of abnormal persistence or regression of embryological veins.
- Recurrent pulmonary embolism following routine infrarenal IVC filter placement should raise suspicion of a duplicated IVC.
- Absent/Interrupted IVC should be suspected in young patients with iliofemoral DVT.
- IVC variants and dilated collateral veins can be mistaken for malignancy.
- Tumour thrombus is differentiated from bland thrombus by filling defect enhancement, vessel lumen expansion and contiguity with the mass.
- Tumour thrombus extent is a key determinant of prognosis and surgical management, particularly in renal cell carcinoma.

## Introduction

The inferior vena cava (IVC) is the main conduit for venous return from the pelvis, abdominal viscera and lower extremities. A comprehensive understanding of IVC anatomy, congenital variants and pathology is instrumental to accurate diagnosis and management. In this review, we discuss normal anatomy, embryogenesis and present illustrated cases of congenital anomalies of the IVC including duplicated IVC, left-sided IVC, absent infrarenal IVC and interrupted IVC with azygos continuation. Diseases involving the IVC can be diagnostically challenging, with computed tomography (CT) and magnetic resonance imaging (MRI) playing key roles in characterising malignancy, tumour extent, caval wall invasion and bland thrombus. This information is crucial for staging and surgical planning. Lastly, we discuss the imaging pitfall of mixed artefact masquerading as thrombus.

### Normal IVC anatomy

The IVC is a large retroperitoneal vein draining the lower extremities, pelvic and abdominal viscera to the right atrium of the heart. It is typically formed by the confluence of the right and left common iliac veins behind the right common iliac artery at the level of the fifth lumbar vertebra. The IVC ascends along the right side of the vertebral column behind the duodenum, portal vein and liver to pierce the diaphragmatic central tendon at the caval opening at the level of the eighth thoracic vertebra. This is followed by a short intra-thoracic course prior to terminating at the right atrium.

As it ascends, it receives many tributaries including paired third and fourth lumbar veins, the right gonadal vein, paired renal veins, the right suprarenal vein, paired inferior phrenic veins and three hepatic veins. There can be variations to these tributaries such as hepatic accessory veins, the right gonadal vein draining into a right renal vein, or a right lumbar azygos vein draining first and second lumbar veins [1–4]. Lastly, the azygos venous system supplies collateral circulation between the superior vena cava (SVC) and IVC.

### IVC embryology

**Fig. 1:**
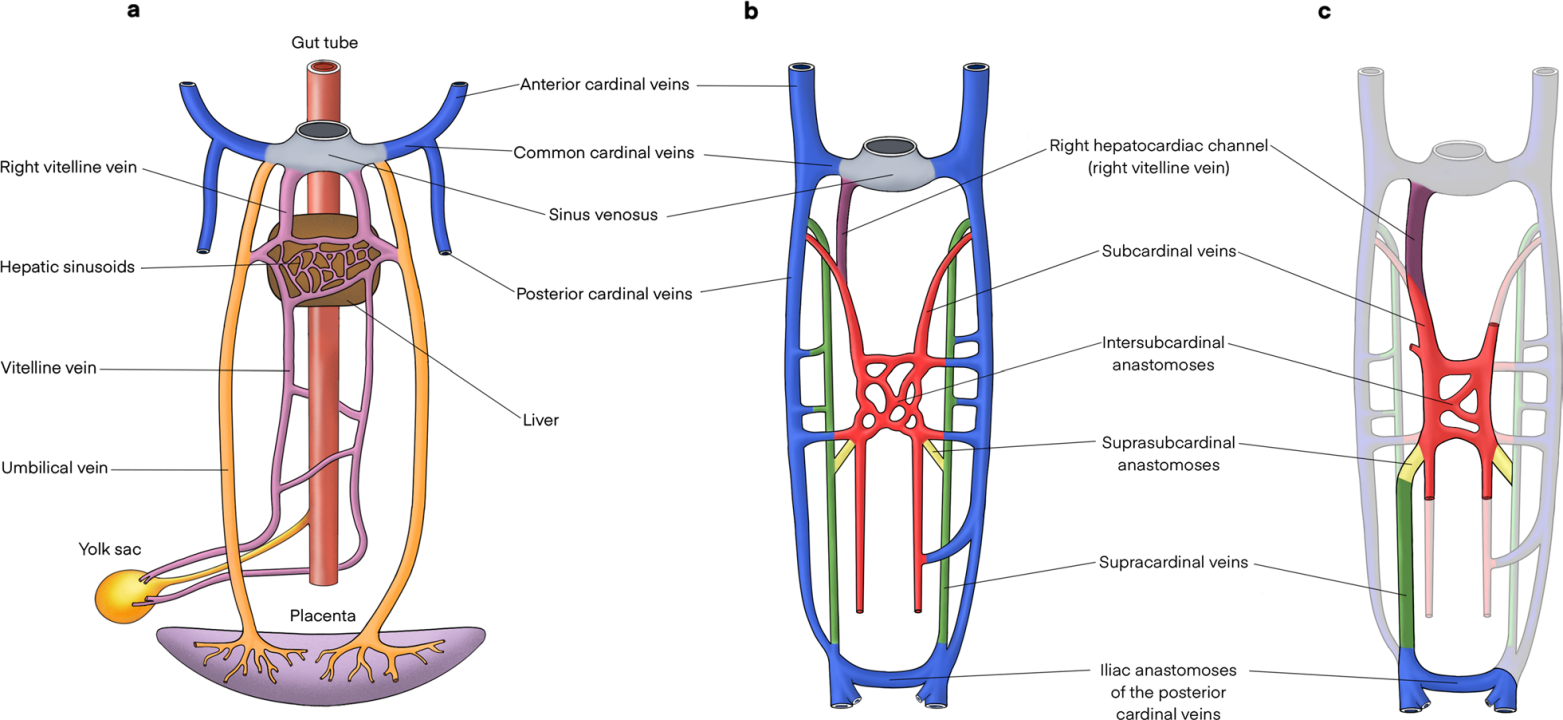
Embryological development of the IVC. **a** The sinus venosus receives paired common cardinal, umbilical and vitelline veins draining blood from general tissue, the placenta and the yolk sac, respectively. **b** The right hepatocardiac channel (purple) is formed by the right vitelline vein and persists to become the hepatic IVC. Paired subcardinal (red) and supracardinal veins (green) emerge and form multiple anastomotic channels. These include the intersubcardinal anastomoses, suprasubcardinal anastomoses and fusion between the right subcardinal vein (red) and developing hepatic IVC (purple). As the embryo matures, some of these anastomoses regress. **c** This illustration highlights vessels that persist to form the mature IVC and its tributaries

The IVC is formed by a complex process of fusion and subsequent regression of embryological veins, namely the posterior cardinal, subcardinal, supracardinal and vitelline veins. In fourth week of embryological development, the right and left horns of the sinus venosus receive paired common cardinal, umbilical and vitelline veins draining blood from general tissue, the placenta and yolk sac, respectively (Fig. 1a) [5, 6].

During the sixth week of gestation, paired posterior cardinal veins are the dominant vessels carrying blood from the caudal portion of the embryo to the common cardinal vein, and later persist as the mature common iliac veins, anastomosing to form the confluence of left and right common iliac veins in the mature venous system [5, 7]. Proximally, paired vitelline and umbilical veins form the hepatic sinusoidal network, including left and right hepatic veins. As the yolk sac and proximal umbilical veins regress, the cranial aspect of the vitelline veins becomes paired hepatocardiac channels, with the right hepatocardiac channel persisting to become the hepatic segment of the IVC [6].

In the following two weeks of foetal development, paired subcardinal and supracardinal veins emerge as dominant tributaries, forming multiple channels draining into the posterior cardinal veins (Fig. 1b) [5]. The subcardinal veins form ventromedial to the posterior cardinal veins, while the supracardinal veins originate dorsomedial to the posterior cardinal veins [7]. Significant anastomotic networks are formed between paired subcardinal veins (intersubcardinal anastomosis) and between supracardinal and subcardinal vessels (suprasubcardinal anastomoses). The proximal posterior cardinal veins subsequently regress, while metanephric kidneys ascend to connect with the suprasubcardinal anastomoses [8]. The intersubcardinal anastomosis forms the left renal vein and in combination with the suprasubcardinal anastomoses contributes to the renal segment of the IVC [9].

At the same time, the cranial end of the right subcardinal vein forms the suprarenal segment of the IVC and fuses with the developing hepatic IVC [9]. Gonadal and suprarenal veins are also derived from the subcardinal veins [6]. The caudal left supracardinal vein and left subcardinal veins regress, establishing right-sided dominance. Paired supracardinal veins and their anastomosis extend above the diaphragm to become the azygos and hemiazygos veins [5, 7]. Caudally, the right supracardinal vein persists as the infrarenal IVC, communicating with the paired iliac veins (Fig. 1c) [6].

**Fig. 2:**
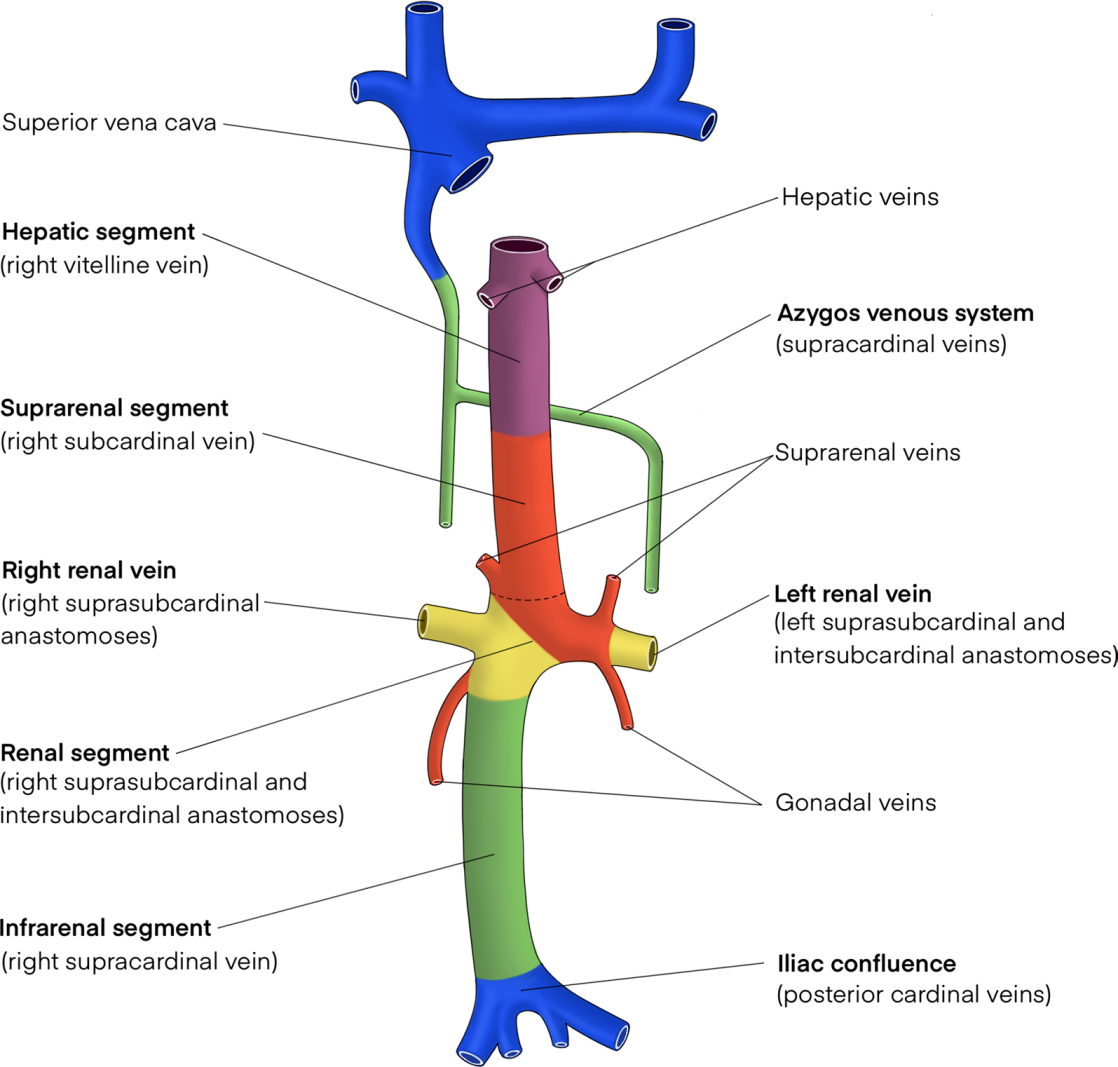
The mature IVC is composed of infrarenal (right supracardinal vein), renal (right suprasubcardinal and intersubcardinal anastomoses), suprarenal (right subcardinal vein) and hepatic (vitelline veins) segments. Renal and suprarenal segments are separated by the dashed line. Posterior cardinal veins persist as paired common iliac veins which join to form the iliac confluence, whilst supracardinal veins contribute to the azygos venous system draining into the superior vena cava. The gonadal and suprarenal veins are derived from subcardinal veins

In summary, the mature IVC is composed of infrarenal (right supracardinal vein), renal (right suprasubcardinal and intersubcardinal anastomoses), suprarenal/infrahepatic (right subcardinal vein) and hepatic (vitelline veins) segments. Posterior cardinal veins persist as paired common iliac veins, whilst the supracardinal veins contribute to the azygos venous system (Fig. 2).

## Anatomical variants

IVC anatomical variants primarily result from abnormal regression or persistence of embryological veins [7]. Although most anomalies are asymptomatic incidental findings, they can cause lower extremity venous insufficiency, deep vein thrombosis, pelvic congestion syndrome and affect planning of vascular procedures [10–17]. The most common anomalies include duplicated IVC, left-sided IVC and interruption of the IVC.

### Duplicated IVC

**Fig. 3:**
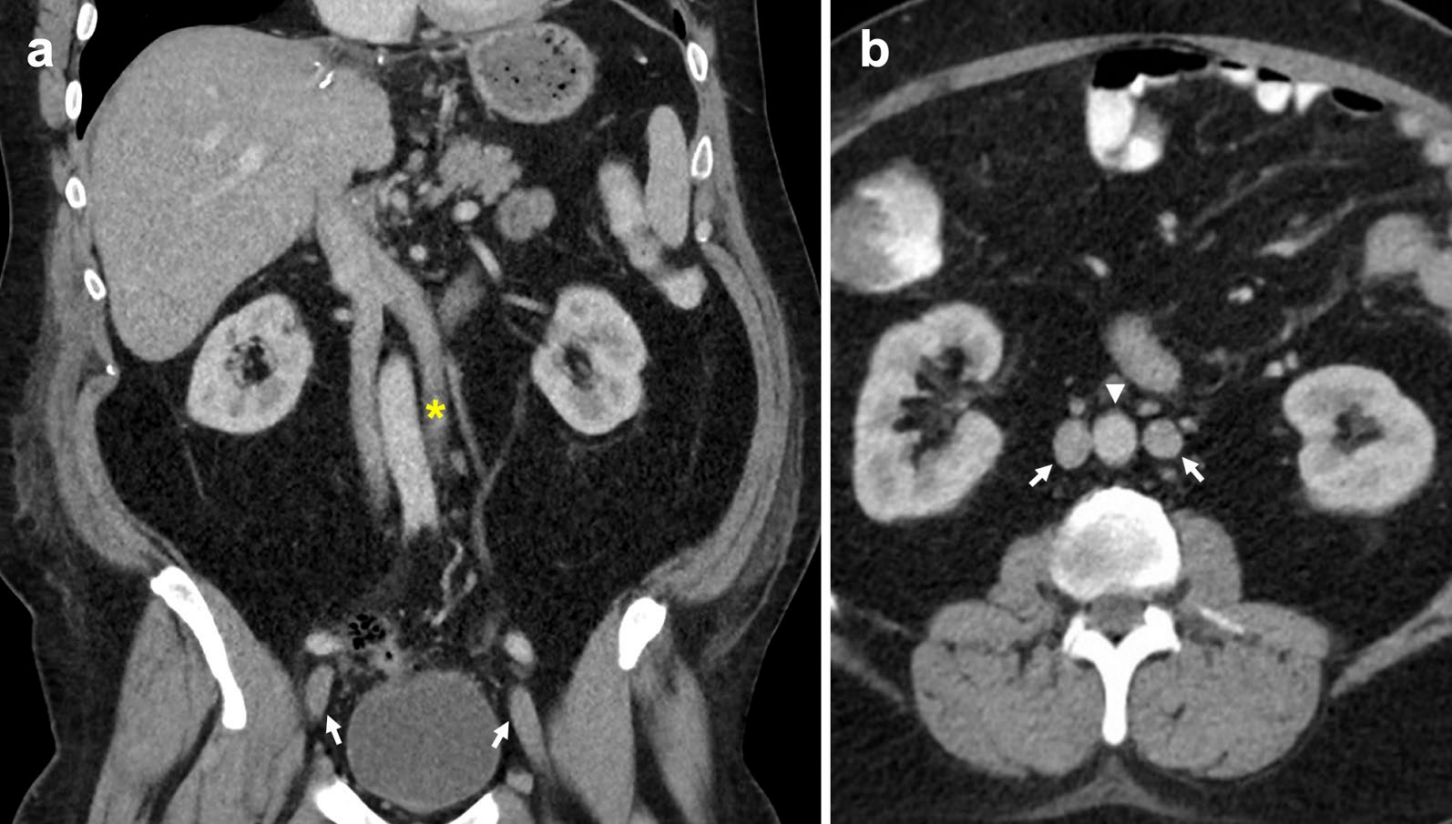
Duplicated IVC with symmetrical caliber. **a** Contrast-enhanced coronal CT shows a duplicated IVC (asterisk) with symmetrical caliber draining into the suprarenal IVC. Bilateral external iliac veins (arrows) both drain into cavae on their respective sides. **b** Axial CT image shows the paired IVC trunks (arrows) positioned on either side of the abdominal aorta (arrowhead)

**Fig. 4:**
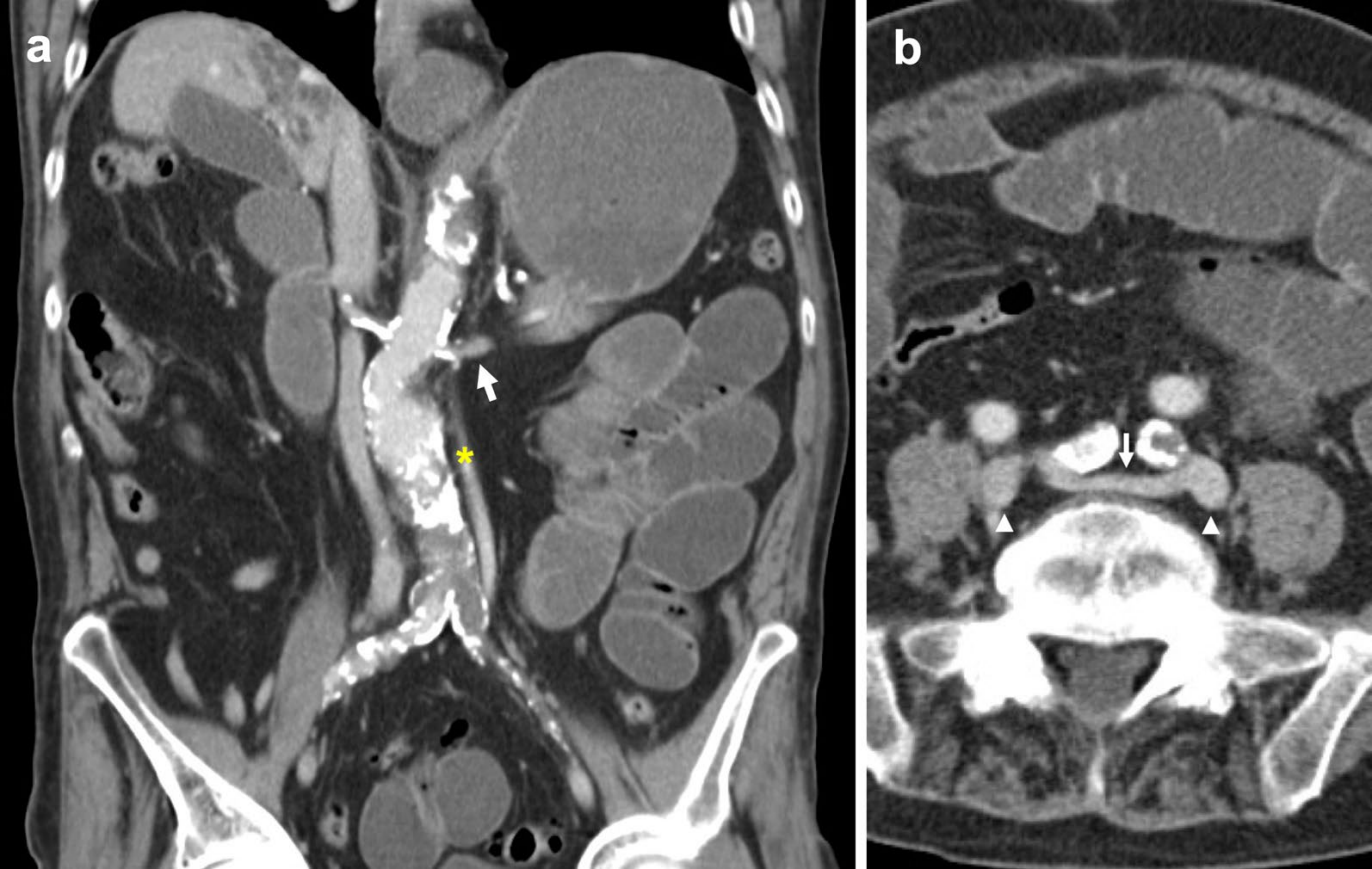
Duplicated IVC with asymmetrical caliber and bridging vein. **a** Contrasted-enhanced coronal CT shows a duplicated IVC (asterisk) of smaller caliber compared to the orthotopic IVC, that approaches and subsequently drains into the left renal vein (arrow). **b** Axial CT image shows a bridging vein (arrow) connecting paired common iliac veins (arrowheads) positioned anterior to the L4 vertebral body and posterior to common iliac arteries

**Fig. 5:**
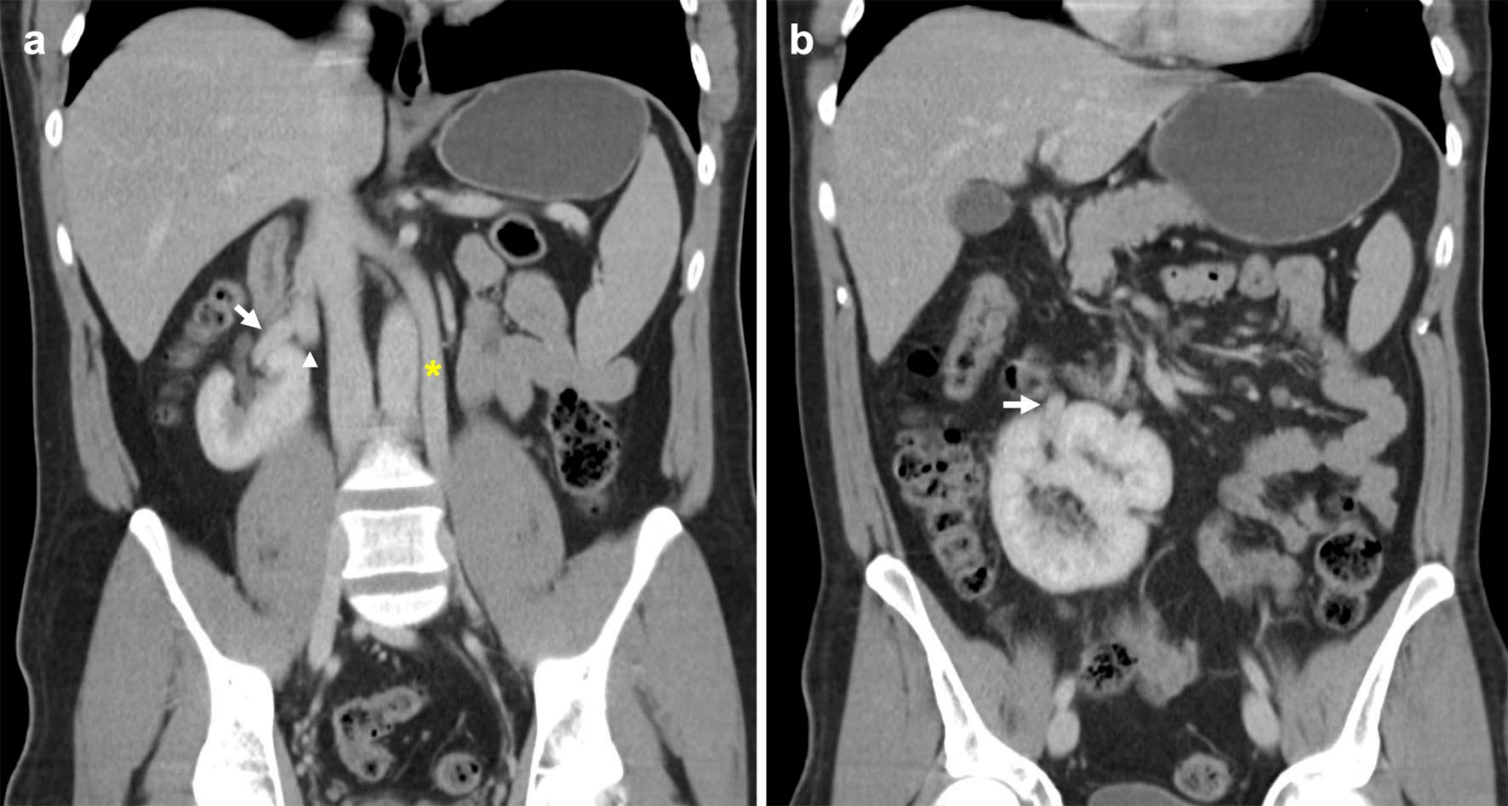
Duplicated IVC with right-sided crossed fused renal ectopia. **a** Coronal CT image shows a duplicated IVC (asterisk) crossing the midline to drain into the right-sided suprarenal IVC. The right renal vein (arrow) and vein of the left ectopic kidney (arrowhead) join before draining into the orthotopic IVC. **b** Coronal CT demonstrates the right-sided crossed fused renal ectopia and its renal vein (arrow)

A duplicated IVC is formed by an abnormal persisting left supracardinal vein, resulting in duplicated infrarenal IVC segments (Fig. 3). This variant has a prevalence of 0.2–3% [18]. The common iliac veins typically drain to their respective sides, with the left infrarenal IVC joining the left renal vein, which in turn crosses to join the orthotopic suprarenal IVC. Some studies subclassify this anomaly by differences in calibre of the duplicated infrarenal IVC segments [19]. In addition, a bridging vein may connect the common iliac veins at the inferior origin of the duplicated IVC (Fig. 4) [20]. Rarely, the duplicated segment can continue as the hemiazygos vein and drain directly into the superior vena cava [21, 22]. Case reports of association with retrocaval ureters, horseshoe kidneys and malrotation of the gut have been described [23–25]. In this review, we present a rarely described case of duplicated IVC and crossed renal ectopia with both renal veins draining into the orthotopic IVC (Fig. 5) [26]. A duplicated IVC can also be mistaken for adenopathy, or a lymph nodes if its tubular nature is not recognised [16, 27]. Lastly, if this anomaly is not recognised, recurrent pulmonary embolism can occur despite routine infrarenal IVC filter placement [28, 29]. Solutions for this scenario include bilateral infrarenal IVC filter placement, suprarenal IVC filter placement, and steel coil embolisation [30–32].

### Left-sided IVC

**Fig. 6:**
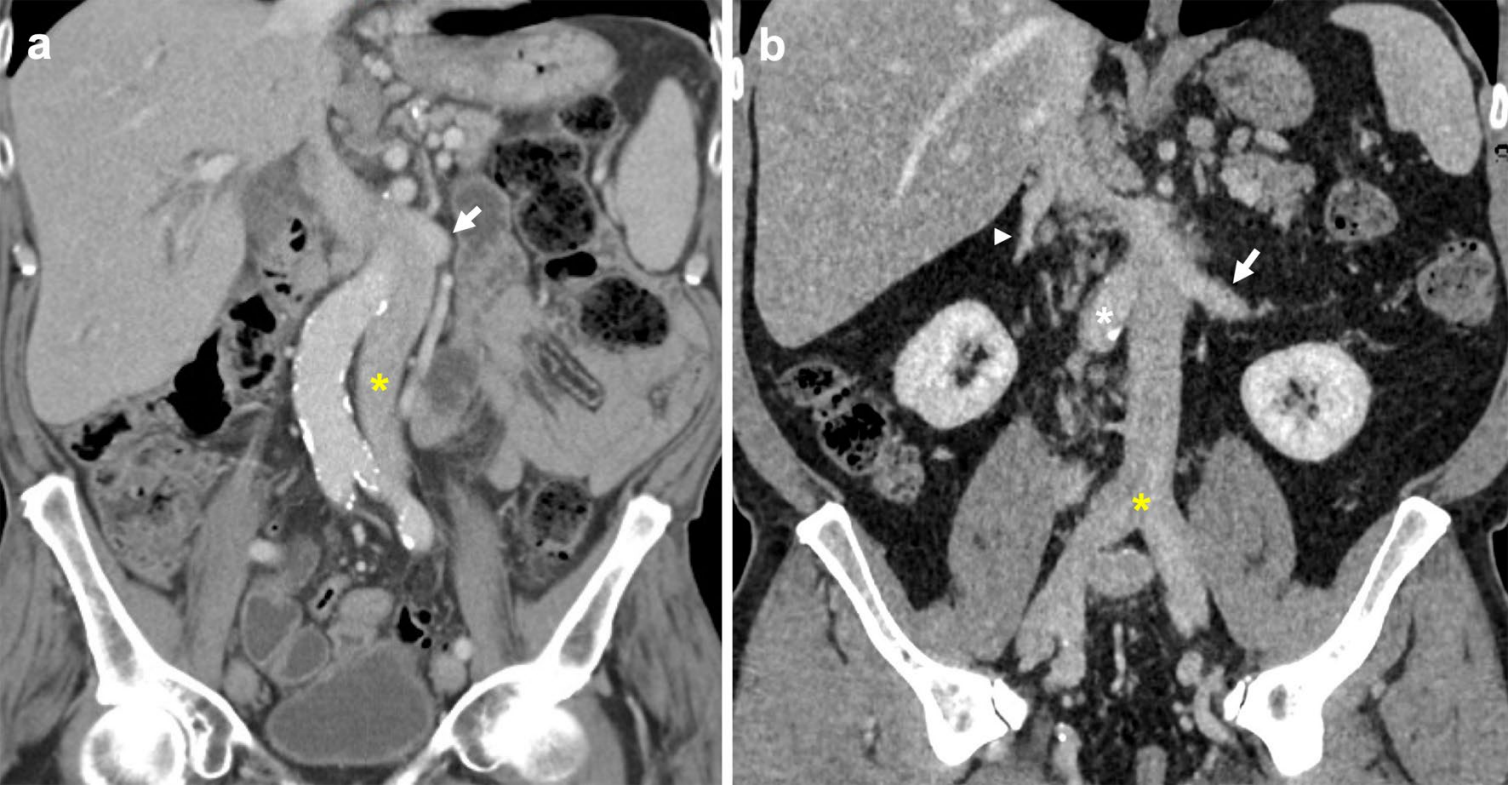
Left-sided IVC. **a** Coronal CT image shows a non-enhanced left-sided IVC (asterisk) draining into an enhanced left renal vein which connects to the hepatic IVC. **b** Coronal CT image of a second case demonstrates a typical confluence of common iliac veins (yellow asterisk) forming the infrarenal IVC on the left side of the aorta (white asterisk) to join the left renal vein (arrow) which drains into the orthotopic IVC. The proximal right renal vein (arrowhead) is also pictured draining into the suprarenal IVC

Abnormal regression of the right supracardinal vein and persistence of the left supracardinal vein results in a left-sided IVC, with a prevalence of 0.2–0.5% [18]. Bilateral common iliac veins drain into the left-sided IVC which typically course superiorly to join the left renal vein (Fig. 6). Known variants to this configuration include hemiazygos continuation of the left-sided IVC, and an associated retroaortic right renal vein [12, 22, 33]. Identifying a left-sided IVC is important as it can complicate procedures such an abdominal aortic aneurysm repair, left-sided nephrectomy, oblique lumbar fusion or IVC filter placement [7, 34–37].

### Absent infrarenal IVC

**Fig. 7:**
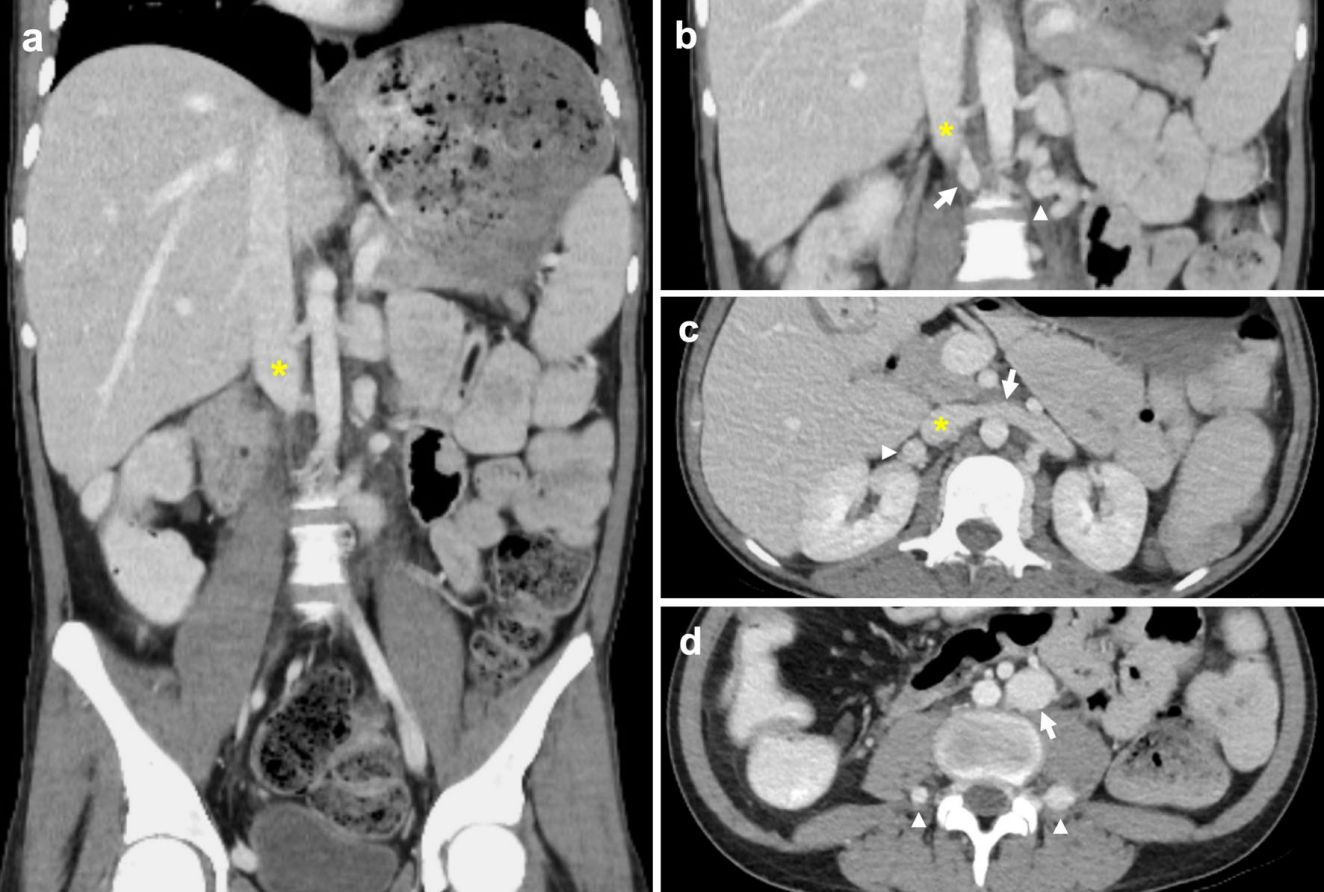
Absent infrarenal IVC with azygos continuation. Paired common iliac veins continue cranially as bilateral ascending lumbar veins. **a** Contrast-enhanced coronal CT demonstrates the caudal end of the IVC (asterisk). **b** Coronal CT shows the right ascending lumbar vein (arrow) approaching and subsequently draining into the IVC (asterisk). Tortuous paravertebral collateral veins (arrowhead) are present adjacent to the aorta. **c** Axial CT image with left renal vein (arrow) crossing anterior to the aorta to drain into the renal IVC segment (asterisk). Right renal vein (arrowhead) also approaches renal IVC segment. **d** Axial CT shows prominent bilateral ascending lumbar veins (arrowheads) and a distended paravertebral collateral (arrow) positioned left of the aorta

**Fig. 8:**
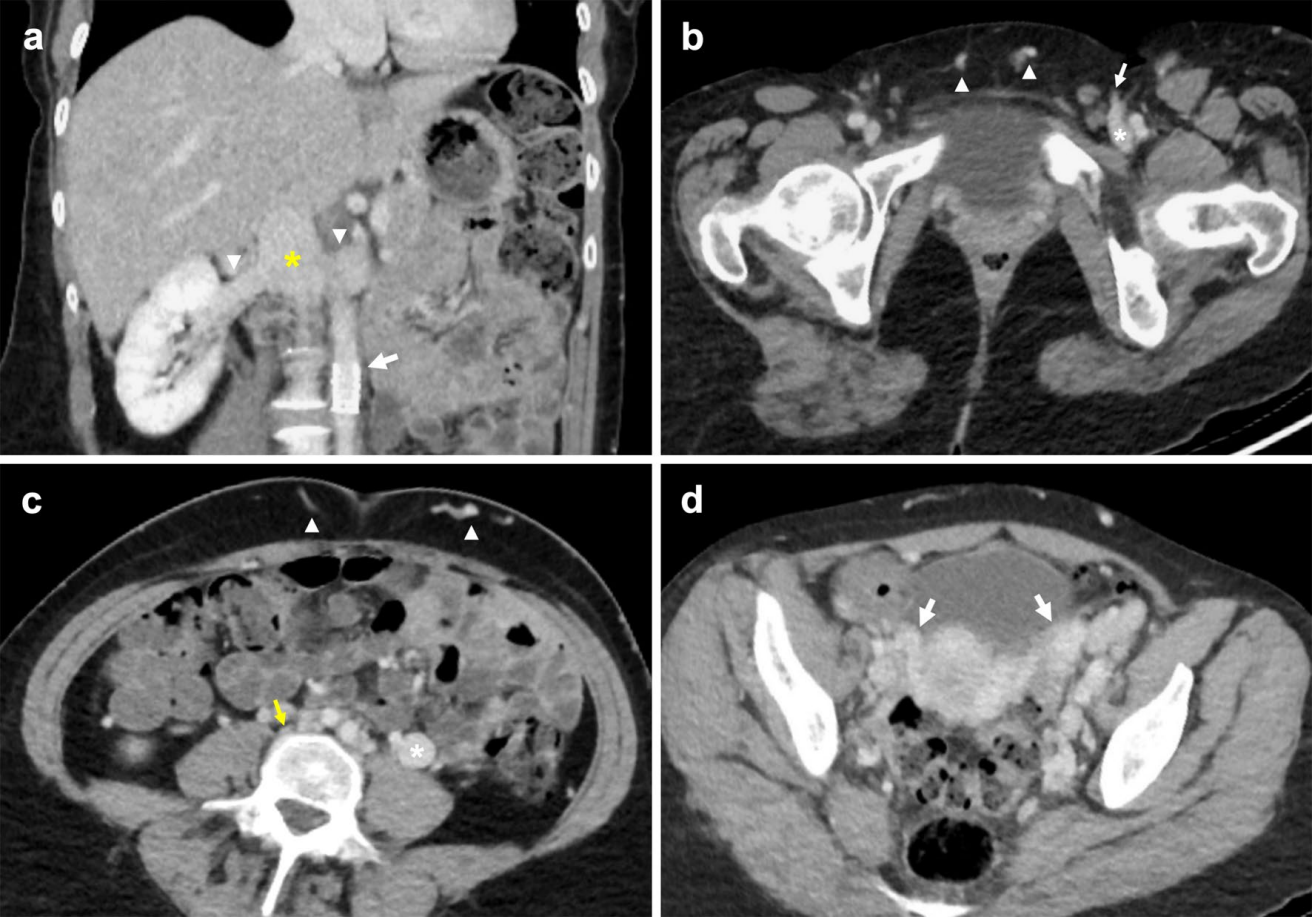
Absent infrarenal IVC with multiple collateral pathways. Contrast-enhanced delayed venous phase on a CT abdomen and pelvis demonstrates prominent superficial abdominal, epigastric and gonadal veins draining the lower extremities. **a** Coronal CT image shows paired renal veins (arrowheads) draining into the caudal end of the IVC (asterisk). An aortic stent (arrow) is visualised inferior to the left renal vein. **b** Axial CT shows prominent external pudendal veins (arrowheads), and the left femoral vein (asterisk) receiving a distended superficial inferior epigastric vein (arrow). **c** Axial CT image with a distended left gonadal vein (asterisk). Small calibre unenhanced infrarenal IVC (arrow) is suggestive of chronic occlusion. Enhanced right inferior epigastric and abdominal wall veins are also visualised (arrowhead). **d** Axial CT demonstrates bilateral enlarged pelvic veins (arrows)

An absent infrarenal IVC, also known as interruption of the infrarenal IVC or infrarenal agenesis of IVC with azygos continuation, is postulated to be caused by acquired intrauterine or perinatal venous thrombosis, rather than failure of embryonic vein development [7, 38, 39]. This leads to failure of posterior cardinal and supracardinal vein development, resulting in external and internal iliac veins draining into the azygos–hemiazygos system via ascending lumbar veins, and a preserved suprarenal IVC segment (Fig. 7) [39, 40]. It is a rarely described anomaly with unknown incidence [11, 39, 41, 42]. Affected patients are at risk of developing lower extremity venous insufficiency, deep vein thrombosis, varicose veins and pelvic congestion syndrome [10–15]. In the absence of adequate flow through ascending lumbar veins and the azygos system, other collateral pathways can form involving abdominal wall, pelvic, gonadal and retroperitoneal vessels (Fig. 8) [13].

### Interrupted IVC with azygos continuation

**Fig. 9:**
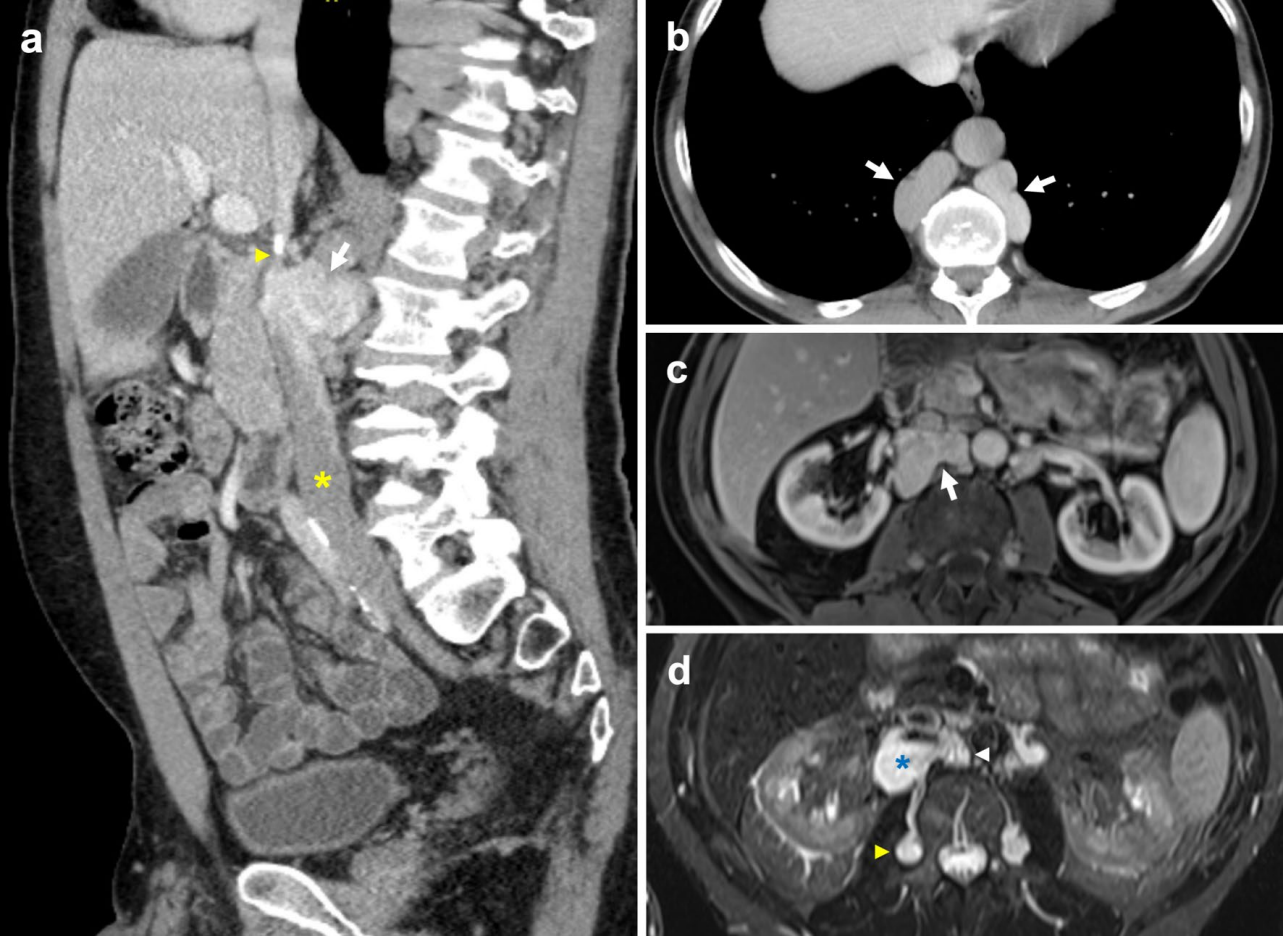
Distended collateral vein mistaken for mass in context of incidentally detected suprarenal IVC stenosis. The 54-year-old man had no previous history of IVC filter insertion or intrabdominal surgery. **a** Contrast-enhanced sagittal CT shows a pseudomass (arrow) immediately posteroinferior to complete stenosis (arrowhead) of the suprarenal IVC, with caudal segments of the IVC (asterisk) preserved. A small focus of calcification is present at the level of the stenosis. **b** Axial CT shows preferential blood flow through markedly distended azygos and hemiazygos veins (arrows). **c** Axial MR T1 VIBE image shows a well-defined lobulated mass (arrow) with venous enhancement. **d** Axial MR T2 image of the mass (asterisk) shows high T2 signal in keeping with slow venous flow. It communicates posteriorly with the right ascending lumbar vein (yellow arrowhead) and medially with a right paravertebral collateral vein (white arrowhead)

Interrupted IVC with azygos continuation classically refers to interruption of the suprarenal/infrahepatic segment and occurs due to failure of the right subcardinal vein to anastomose with the vitelline vein [7, 43]. An interrupted IVC has been described in various ways with ‘interrupted’ being interchanged for absence, anomalous or agenesis [13, 22, 44]. The suprarenal IVC reroutes to drain via the azygos vein, while the hepatic IVC only receives the hepatic veins. It carries a prevalence of 0.6% and is classically associated with polysplenia, cardiovascular malformations and situs anomalies [7, 16, 45]. Like an absent infrarenal IVC, an interrupted IVC without adequate collateral pathways can similarly result in vascular problems such as deep vein thrombosis and venous insufficiency. An enlarged azygos vein can be misinterpreted as retrocrural lymphadenopathy or a right paratracheal mass, while a distended hemiazygos vein may simulate a left-sided mediastinal mass [43, 46, 47]. Prominent collateral vessels can also be mistaken for paraspinal masses (Fig. 9) [42]. Preoperative awareness of this anomaly is important prior to thoracic and cardiopulmonary bypass surgery [48, 49].

**Fig. 10:**
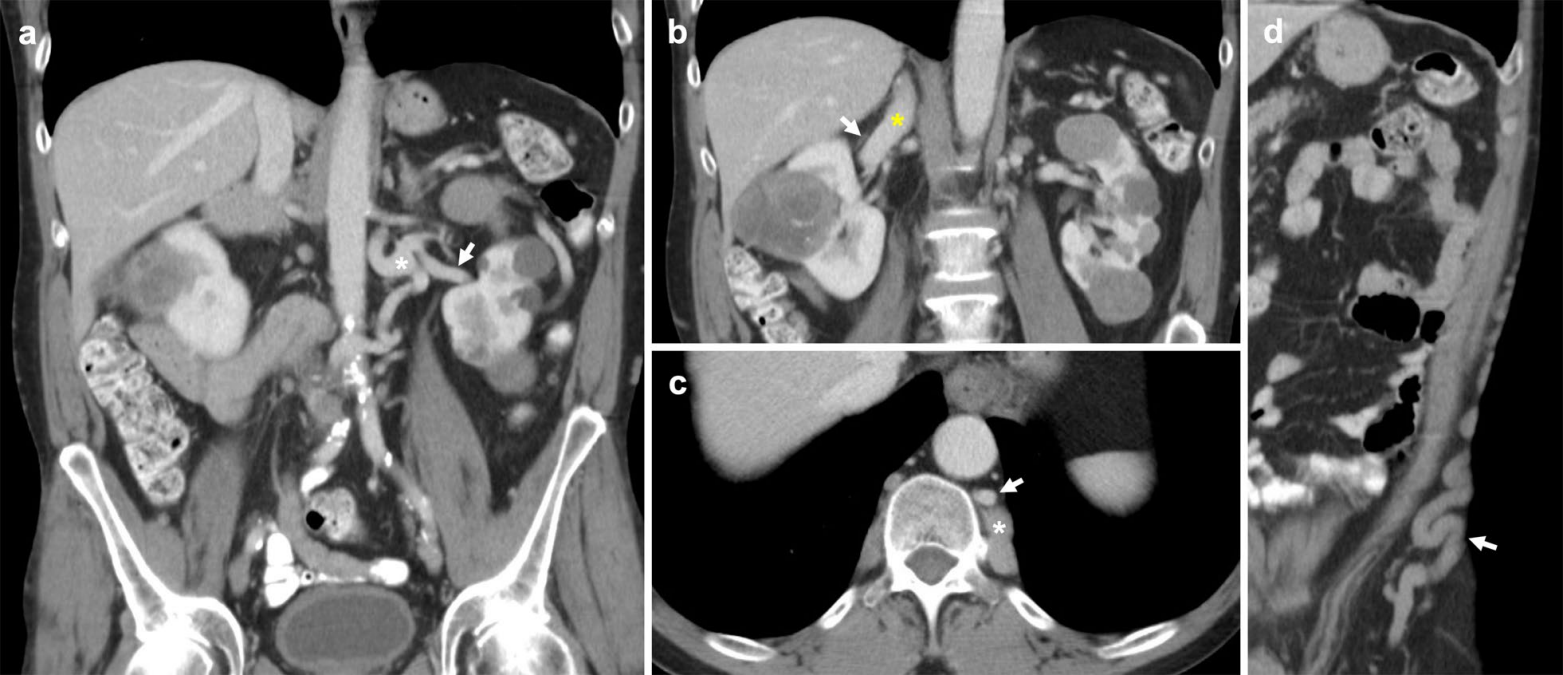
Interrupted IVC (renal level) with azygos and hemiazygos continuation. Anomaly detected incidentally on imaging for a 55-year-old man with biopsy proven renal cell carcinoma. The paired common iliac veins continue as bilateral ascending lumbar veins draining into the azygos–hemiazygos system. **a** Contrast-enhanced coronal CT shows the left renal vein (arrow) draining into a tortuous paravertebral collateral (asterisk). **b** The right renal vein (arrow) joins the suprarenal IVC (asterisk). **c** Axial CT image depicts a left-sided paravertebral collateral (arrow) that drains into the hemiazygos vein (asterisk) sited posteriorly. **d** Coronal CT image with a prominent lateral abdominal wall collateral vein

Koc et al. uses a straightforward nomenclature that identifies all anomalies with absent or interrupted segments as ‘interrupted IVC’, followed by the level of interruption and associated collaterals, i.e. ‘interrupted IVC (suprarenal level) with azygos continuation’ [12]. In this review, we report a rarely described case of interrupted IVC of the renal segment with azygos continuation (Fig. 10) [13]. Instead of draining directly into the IVC, the left renal vein drains into a tortuous paravertebral collateral that eventually joins the hemiazygos vein, while the right renal vein continues to drain directly into the IVC. Paired common iliac veins continue as bilateral ascending lumbar veins to join a distended azygos–hemiazygos system. Lastly, acquired pathology such as complete stenosis of the suprarenal IVC results in anatomy similar to a congenital interrupted IVC, with development of collateral pathways and preferential blood flow into the paravertebral, azygos and hemiazygos systems (Fig. 9).

## Acquired pathologies

**Fig. 11:**
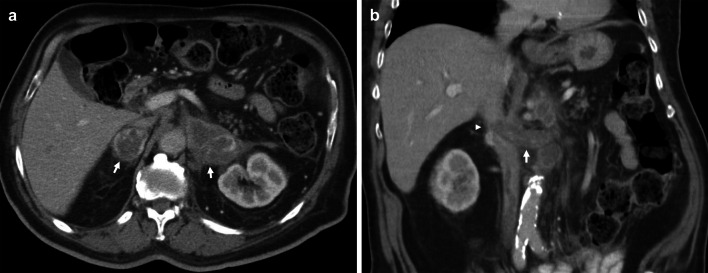
Adrenal metastases with suprarenal IVC tumour thrombus in a patient with small cell lung carcinoma primary. **a** Axial CT shows bilateral suprarenal lesions with heterogeneous peripheral enhancement in keeping with bilateral adrenal metastases. **b** Coronal CT demonstrates extension of tumour into the left renal vein (arrow) and suprarenal IVC (arrowhead). The heterogeneous appearance of the suprarenal IVC is due to a combination of tumour thrombus and flow artefact

**Fig. 12:**
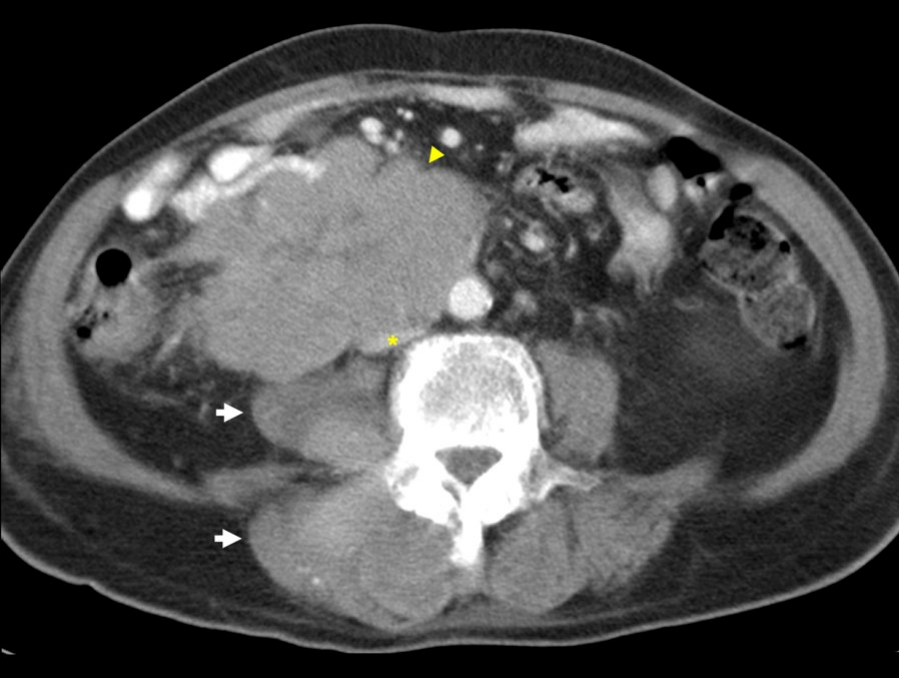
Extrinsic compression of IVC. Contrast-enhanced axial image demonstrating extrinsic compression of the IVC (asterisk), which is flattened, secondary to a large nodal mass (arrowhead) from lymphoma. Further lymphomatous infiltration of the right psoas and paravertebral muscles is also present (arrows)

Acquired conditions affecting the IVC include primary and secondary malignancy with or without intravascular extension, benign tumours, extrinsic compression, bland thrombus, and chronic obstruction. Primary IVC malignancy is rare, representing less than 1% of all malignancies [50, 51]. In contrast, secondary IVC malignancy is much more common and often results from tumour thrombus, i.e. direct intravascular extension from an abdominal primary such as kidney, liver or adrenal gland [16]. Metastatic lesions may also invade the caval wall, the most common of which are liver metastases from colorectal cancer [52, 53]. Lung carcinoma involving the IVC is extremely rare, and in this review, we present a case of adrenal metastases from non-small cell lung carcinoma (NSCLC) with extension into the suprarenal IVC (Fig. 11) [54]. Tumours such as pheochromocytoma and leiomyomas are typically benign; however, there are reports of IVC invasion and metastasis [55, 56]. The IVC may also be compressed extrinsically by tumours such as lymphoma, causing symptoms of IVC syndrome such as lower limb and lower torso swelling (Fig. 12). Furthermore, distinguishing tumour from bland thrombus is important to guide anticoagulation therapy [57]. Enhancement of the filling defect, expansion of the vessel lumen, contiguity with mass and increased FDG uptake on PET-CT are findings characteristic of tumour thrombus [58, 59]. Compared to CT, MRI demonstrates superior characterisation of thrombus composition, identifying a greater proportion of tumour thrombi [60]. Finally, determining the extent of IVC involvement affects staging and is critical to surgical planning.

## Primary malignancy

### IVC Leiomyosarcoma

**Fig. 13:**
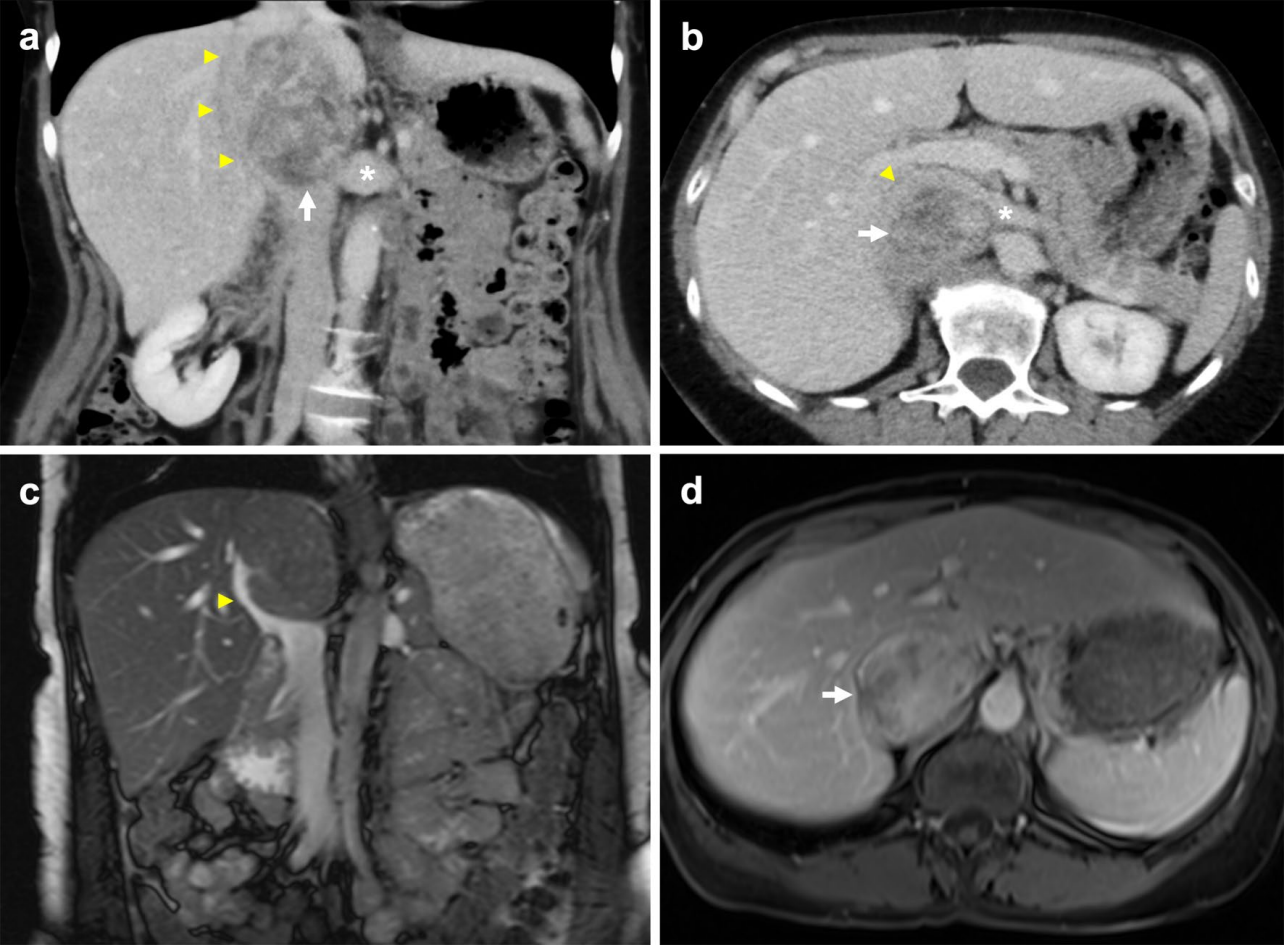
IVC Leiomyosarcoma mistaken for adrenal lesion. **a,b** Coronal and axial contrast-enhanced CT demonstrate a heterogeneous mass (white arrows) displacing the left renal vein (asterisks) anteriorly and hepatic cava (yellow arrowheads) anterolaterally to the right. **c** T2-weighted axial MR image shows the effacement of the hepatic cava (arrowhead) without invasion of adjacent structures. **d** Contrast-enhanced T1-weighted fat-saturated axial image demonstrates heterogeneous enhancement of the tumour (arrow)

IVC leiomyosarcoma is the most common primary malignancy of the IVC and the most common type of retroperitoneal leiomyosarcoma [61]. It is a rare slow-growing smooth muscle cell malignancy that usually occurs in middle-aged women [50, 62–64]. Whilst initial growth is intramural, two-thirds of tumours will eventually demonstrate extraluminal growth [63–65]. Intraluminal tumours can cause venous obstruction, whilst extraluminal tumours can be confused with secondary malignancy from surrounding organs (Fig. 13) [66].

Whilst amenable to wide local excision, it carries a poor prognosis with studies showing 94% recurrence at 5 years and 14% survival at 10 years [62–64, 67]. The level of IVC involvement is an important prognostic factor and can be divided by infrarenal (34–44%), suprarenal (42–50%) and suprahepatic (6–24%) involvement, with 21–36% of cases involving two or more segments [63, 68, 69]. Renal and suprarenal IVC leiomyosarcomas (42–50%) carry the most favourable prognosis, whilst suprahepatic tumours have the worst outcomes due to poor surgical resectability [62, 65].

**Fig. 14:**
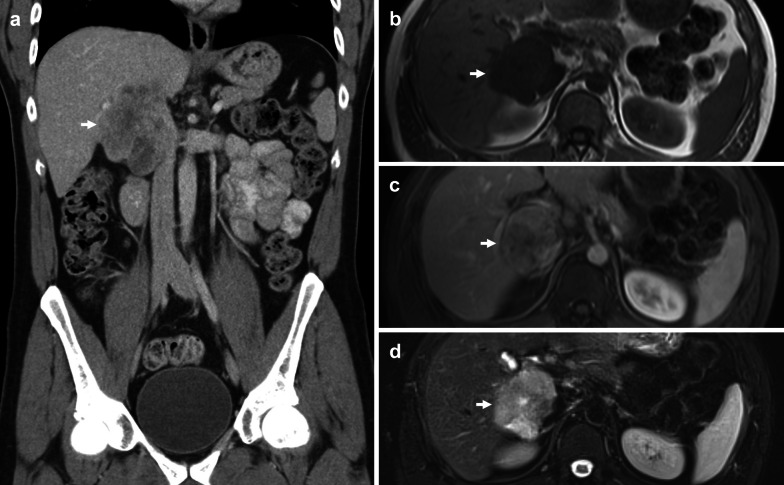
IVC Leiomyosarcoma. **a** Coronal CT image shows a large lobulated heterogeneously enhancing mass (arrow) between the liver and right kidney. It demonstrates both intra and extraluminal growth. On axial MRI, the lesion (arrows) is (**b**) homogeneously T1 hypointense to skeletal muscle, (**c**) enhances heterogeneously, and (**d**) is heterogeneously T2 hyperintense. Some non-enhancing areas are bright on T2 weighted sequences, suggestive of cystic or necrotic components. MRI also demonstrates a positive embedded organ sign

A key characteristic of this tumour is a retroperitoneal mass limited to the IVC. Nishino et al. describe the positive embedded organ sign which is useful for identifying masses that arise from plastic organs such as a vein (Fig. 14) [70]. In this case, IVC leiomyosarcoma will appear embedded in the vessel wall. An imperceptible caval lumen is another specific sign for IVC leiomyosarcoma, in which signal from the IVC is imperceptible at the point of contact between the tumour and the IVC [71]. On both CT and MRI, the mass can demonstrate heterogeneous enhancement due to central cystic or necrotic components (Fig. 13) [72, 73].

## Secondary malignancy

### Renal cell carcinoma

Renal cell carcinoma (RCC) is the most common secondary malignancy of the IVC, with 4–10% of cases demonstrating intravascular spread; an independent prognostic factor for decreased survival rates [74, 75]. RCC tumour thrombus shows little tendency to invade vessel walls; however, when it occurs, it also worsens prognosis [76, 77]. Despite this, complete resection of non-metastatic RCC with IVC extension carries a reasonable 5-year survival rate of 37–68% [78, 79].

Although some practices continue to use the Robson staging system for RCC, it has largely been replaced by the tumour–node–metastasis (TNM) classification system of malignant tumours. Stages T3a, T3b and T3c correspond to renal vein, infradiaphragmatic and supradiaphragmatic IVC extension, respectively, with invasion of the IVC wall also considered to be stage T3c [80]. Increasing venous tumour thrombus extension is negatively associated with long-term survival and alters surgical management [79, 81, 82]. A multi-institutional study of 1122 patients showed a 5-year survival of 22% for stage T3c disease compared to 43.2% for T3a disease [79].

**Fig. 15:**
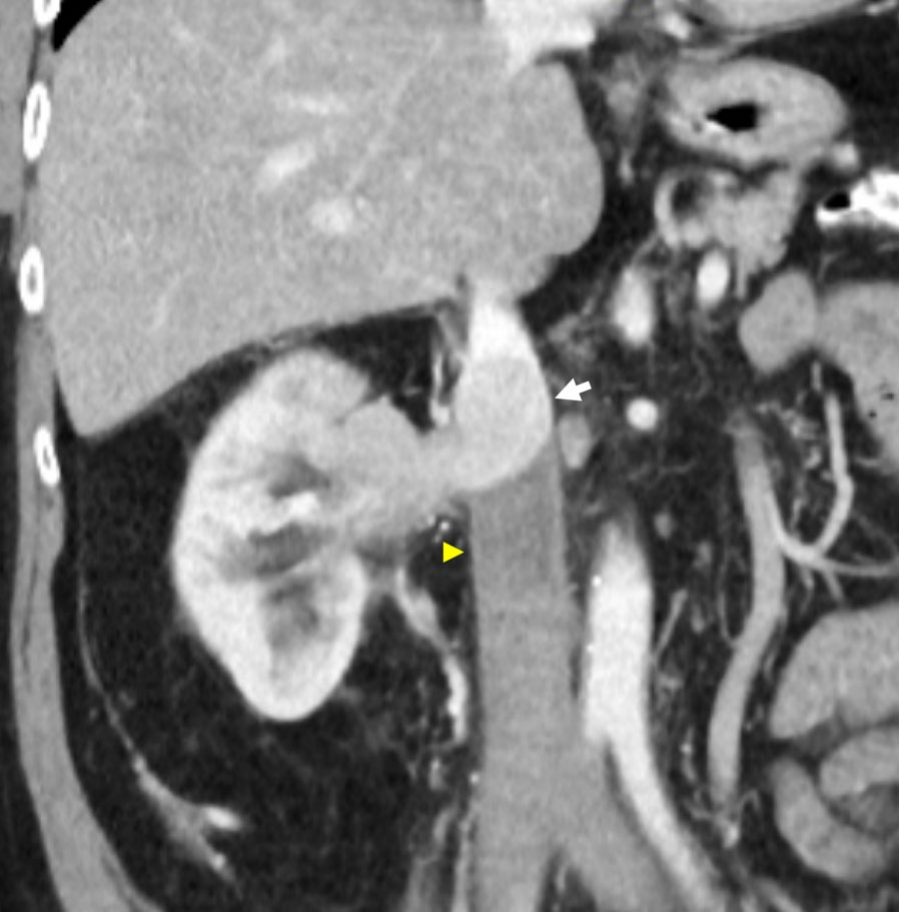
Renal cell carcinoma with Mayo level II tumour thrombus and infrarenal bland thrombus. Coronal CT demonstrates occlusive enhancing tumour thrombus (arrow) extending from the right renal vein into the suprarenal IVC. Infrarenal IVC (arrowhead) and iliac vessels show no contrast enhancement and are expanded in keeping with bland thrombus

**Fig. 16:**
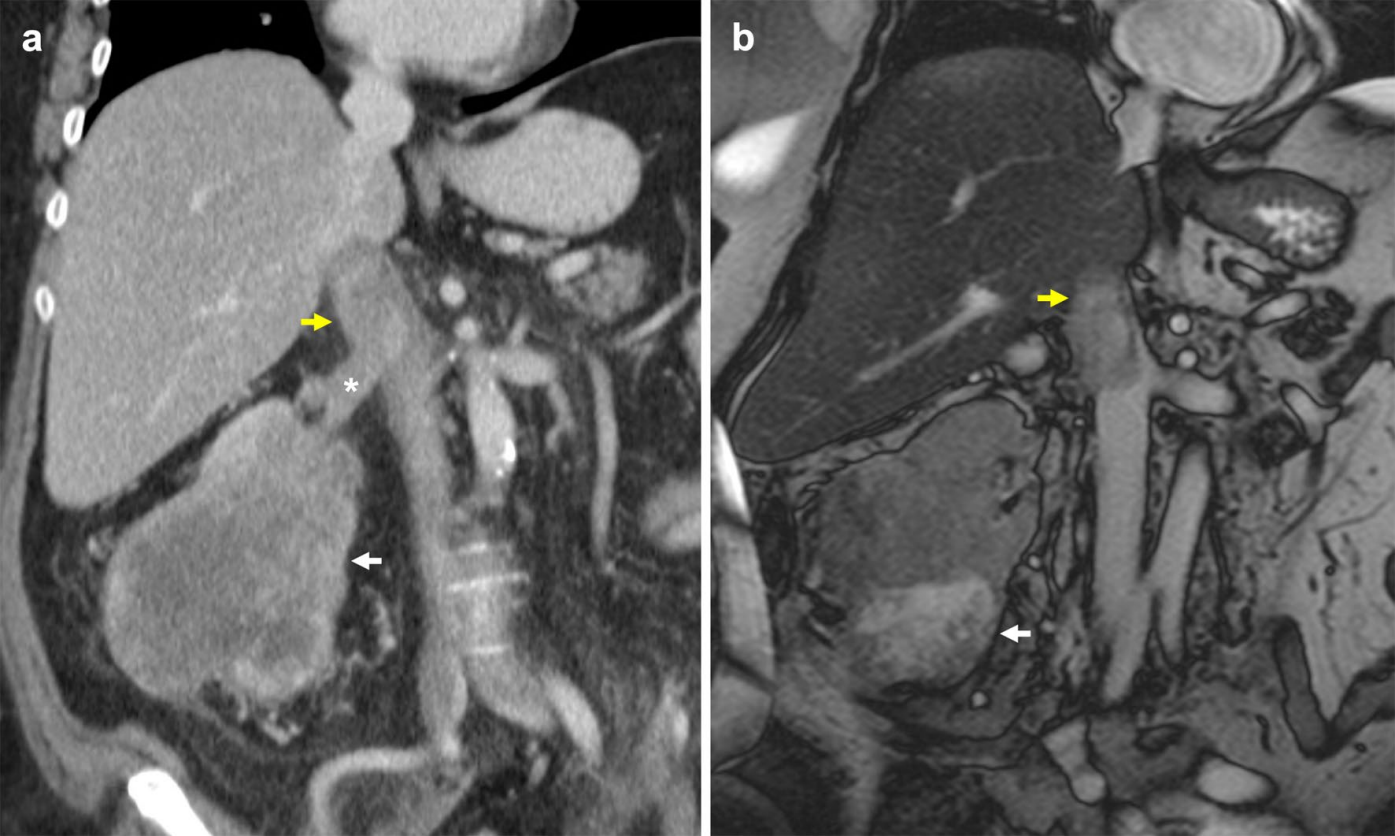
Renal cell carcinoma with Mayo level III thrombus. **a** Coronal CT shows a large heterogeneous renal mass (white arrow), expanded right renal vein (asterisk) and heterogeneous enhancement extending into the suprarenal IVC (yellow arrow) in keeping with tumour thrombus (**b**) T2-weighted coronal image confirms extension of tumour thrombus into the suprarenal and intrahepatic IVC (yellow arrow), which returns similar heterogeneous T2 signal as the primary renal cell carcinoma (white arrow)

The most frequently used classification for RCC tumour thrombus is the Mayo system introduced by Neves and Zincke which stratifies IVC thrombus into 4 levels; level I extending < 2 cm from the renal ostia, level II extending > 2 cm but below the intrahepatic vena cava (Fig. 15), level III involving the intrahepatic IVC (Fig. 16), and level IV being supradiaphragmatic [83]. Mayo level I tumours are generally easy to milk into the renal vein, level II tumours require more extensive vena cava dissection, and level III/IV tumours are associated with higher early complications and often require veno-venous or cardiopulmonary bypass [84].

Both CT and MRI show high accuracy for detecting tumour thrombus, assessing its extent and distinguishing it from bland thrombus (Fig. 15) [85–87]. CT demonstrates 96% accuracy in the corticomedullary phase and allows for simultaneous imaging for metastatic disease [86, 88]. Whilst there has not been a systematic comparison between CT and MRI for detecting IVC wall involvement, both modalities have been shown to reliably predict wall invasion which requires more complicated surgery involving IVC resection. Adams et al. showed complete venous occlusion or vessel breach (tumour signal on both sides of the vessel wall) on MRI can reliably predict intraoperative wall adherence [89]. Moreover, Psutka et al. demonstrated imaging features such as a right-sided RCC, complete IVC occlusion at the right renal vein ostium and anteroposterior IVC diameter ≥ 24 mm at that same level were associated with an increased chance of IVC resection [87]. Lastly, IVC wall invasion can be confidently excluded if tumour thrombus does not contact the vessel wall [89].

Patients with adherent tumour thrombus invading the IVC wall require en bloc excision, including segmental resection and reconstruction of the vessel, and tumour thrombectomy. Whilst the majority of radical nephrectomies with venous thrombectomy are performed by open surgery, there is an increasing trend towards minimally invasive laparoscopic and robotic approaches [81].

### Hepatocellular carcinoma

**Fig. 17:**
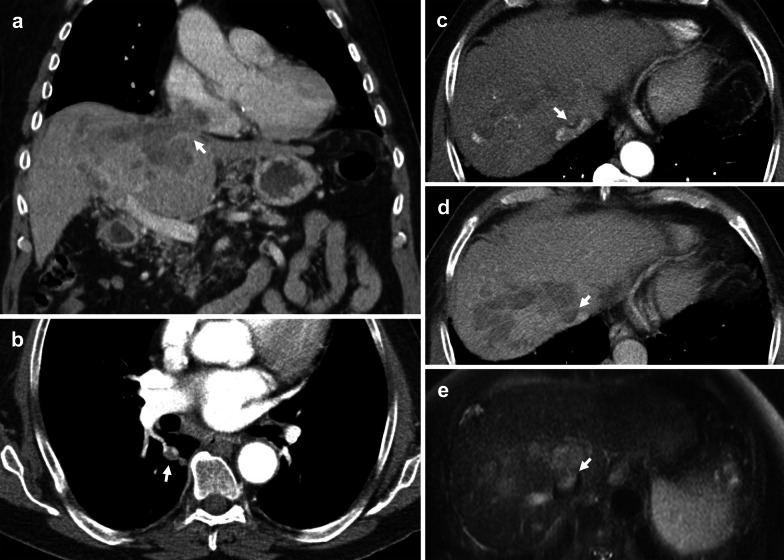
Hepatocellular carcinoma with IVC tumour thrombus, direct invasion of the right atrium and embolic tumour thrombus. **a** Contrast-enhanced coronal CT depicts direct invasion of the HCC (arrow) into the right atrium. **b** Axial CT chest shows subsegmental right pulmonary artery dilatation with internal filling defect (arrow), likely due to embolic tumour thrombus. **c** Axial CT shows multifocal arterial enhancement in the liver, predominantly in segment 7 and 8. The IVC tumour thrombus also demonstrates arterial enhancement (arrow), **d** is visualised as a partial filling defect (arrow) in portal venous phase, and **e** shows intermediate T2 signal (arrow) with extension into the hepatic IVC via the middle hepatic vein

Hepatocellular carcinoma (HCC) typically invades the portal venous system; however, involvement of the IVC is not uncommon, occurring in 4.0–5.9% of patients either through extension of hepatic vein tumour thrombus or direct wall invasion [61, 90]. Tumour thrombus is uncommon in other hepatic malignancies and therefore, should be considered a differentiating feature of HCC [91]. IVC and right atrial involvement carries an extremely poor prognosis due to predisposition to metastatic disease, carrying a median survival of 1–5 months [92–94]. Hepatic venous outflow obstruction may cause Budd–Chiari syndrome and clinical manifestations of portal hypertension [95]. Tumour thrombus may also embolise and obstruct pulmonary arteries (Fig. 17) [96].

**Fig. 18:**
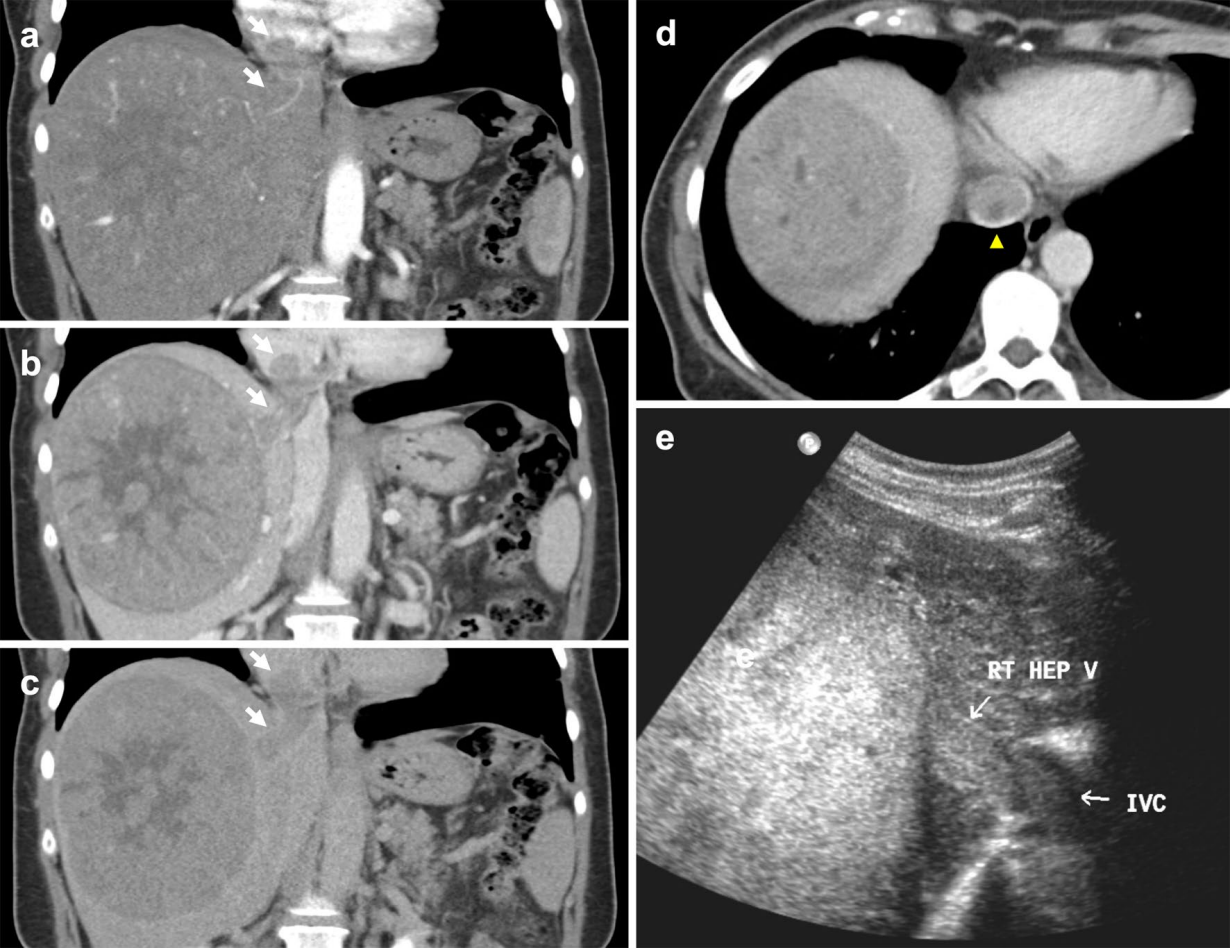
Hepatocellular carcinoma invading the IVC. **a–c** Coronal multiphase CT images show a 12 × 11 cm necrotic hepatic lesion invading the hepatic and supradiaphragmatic IVC. The thrombus (arrows) demonstrates enhancement in both (**a**) arterial and (**b**) portal venous phases, with complete washout in the (**c**) delayed phase. **d** Axial CT shows near complete obstruction of the hepatic IVC (arrowhead). **e** US Abdomen demonstrates a hyperechoic hepatic mass extending into the right hepatic vein and IVC

Most studies focus on imaging findings of portal vein tumour thrombus; however, parallels may be drawn when identifying IVC tumour thrombus. On CT, HCC tumour thrombus demonstrates hepatic vein expansion and arterial enhancement with washout in the portal vein and delayed phases (Fig. 18). Catalano et al. have shown that diffusion-weighted imaging may be employed to differentiate bland thrombus from tumour extension, with bland thrombus eliciting higher ADC values [97]. Patients typically have concomitant liver dysfunction, poor prognosis and high rate of recurrence; therefore, HCC with IVC invasion is considered an absolute contraindication to liver transplantation and managed non-surgically. However, recent studies have shown that sorafenib, external beam radiotherapy and surgery with careful patient selection can improve clinical outcomes [98, 99].

### Adrenal cortical carcinoma

**Fig. 19:**
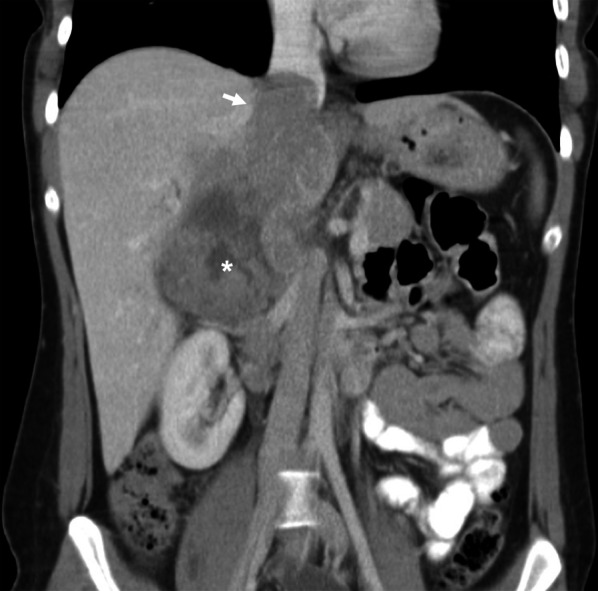
Adrenal cortical carcinoma invading IVC. Portal venous CT coronal image demonstrates a large adrenal cortical carcinoma (asterisk) with irregular margins, areas of necrosis and invasion of the IVC (arrow)

Adrenal cortical carcinoma (ACC), also known as adrenocortical carcinoma, is a rare and aggressive malignancy with an incidence of approximately 1 to 2 in a million, and bimodal age distribution with peaks at under 5 years of age and the 4th to 6th decades of life [100, 101]. Rate of local recurrence is high, and half of adult patients present with advanced disease [101]. 62% of cases are reported to be endocrinologically functional, manifesting as Cushing’s syndrome, Conn’s syndrome, feminisation or virilisation secondary to elevated androgens [101]. Most ACC tumours are larger than 6 cm, have irregular margins and enhance heterogeneously on both CT and MRI due to central areas of necrosis and haemorrhage (Fig. 19) [102, 103]. The presence of T1 hypointensity, T2 hyperintensity and heterogeneous signal drop on chemical shift imaging further supports diagnosis of ACC [103–105]. Moreover, they demonstrate contrast retention on delayed contrast-enhanced CT, with a relative percentage washout of less than 40% [106]. Invasion into the IVC is classified as Stage III disease and occurs in 9–19% of cases [101, 107]. One study found tumour thrombus was more common in right-sided ACC and tumours larger than 9 cm [86].

### Pheochromocytoma

**Fig. 20:**
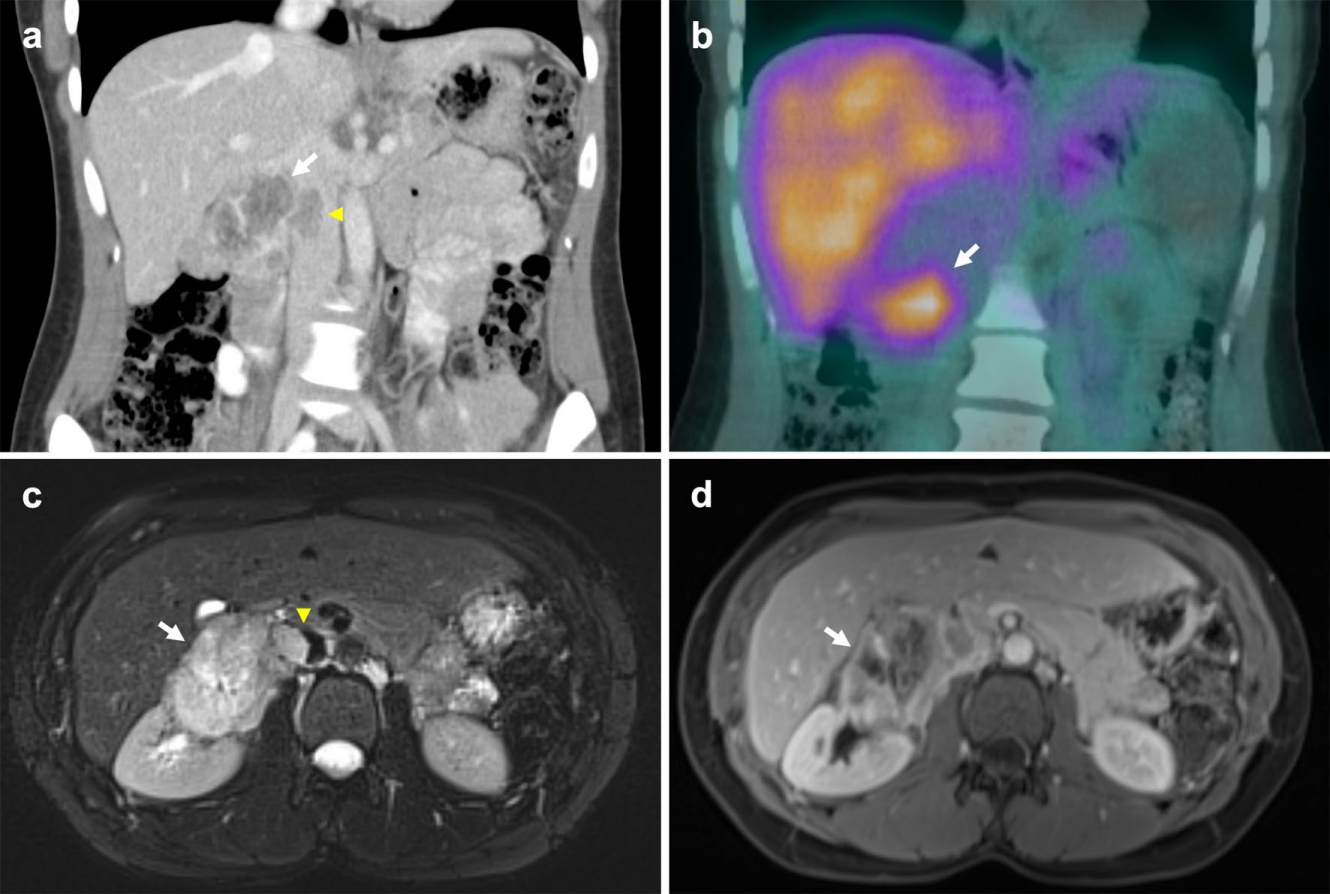
Malignant extra-adrenal phaeochromocytoma invading IVC. **a** Coronal portal venous phase CT shows a heterogeneously enhancing peri-renal mass (arrow) with possible central necrosis that abuts and extends into the infrarenal IVC (arrowhead). **b** SPECT CT demonstrates a focus of abnormal MIBG uptake (arrow) within the region of the peri-renal mass. Selected MR images show the retroperitoneal lesion (**c**) partially obstructing the IVC (arrowhead), demonstrating heterogeneous elevated T2 signal (arrow) on the selected T2-weighted fat-saturated axial image and (**d**) heterogeneous enhancement (arrow) on the selected T1-weighted fat-saturated axial image

Pheochromocytoma is a rare tumour that typically originates from chromaffin cells of the adrenal medulla. 15–20% of these are extra-adrenal, arising from paraganglion chromaffin tissue of the nervous system and are also known as paragangliomas [108]. Known associations include familial syndromes such as von Hippel–Lindau disease, MEN 2A and 2B and neurofibromatosis type 1 [109]. Patients may present with back pain and symptoms consistent with catecholamine secretion [110]. Although pheochromocytomas are usually benign, 32–52% of all extra-adrenal pheochromocytomas are malignant with an increased propensity to metastasise and invade local structures such as the caval wall (Fig. 20) [111–113]. These tumours typically enhance heterogeneously with delayed washout on both CT and MRI and may show marked T2 hyperintensity [114, 115]. When ruling out metastases, CT in combination with functional imaging such as ^18^F-FDA or ^123^I-MIBG may be employed [116]. Surgical resection is the treatment of choice with pre-operative blood pressure control required to prevent intraoperative hypertensive crises [117].

### Thrombosis

**Fig. 21:**
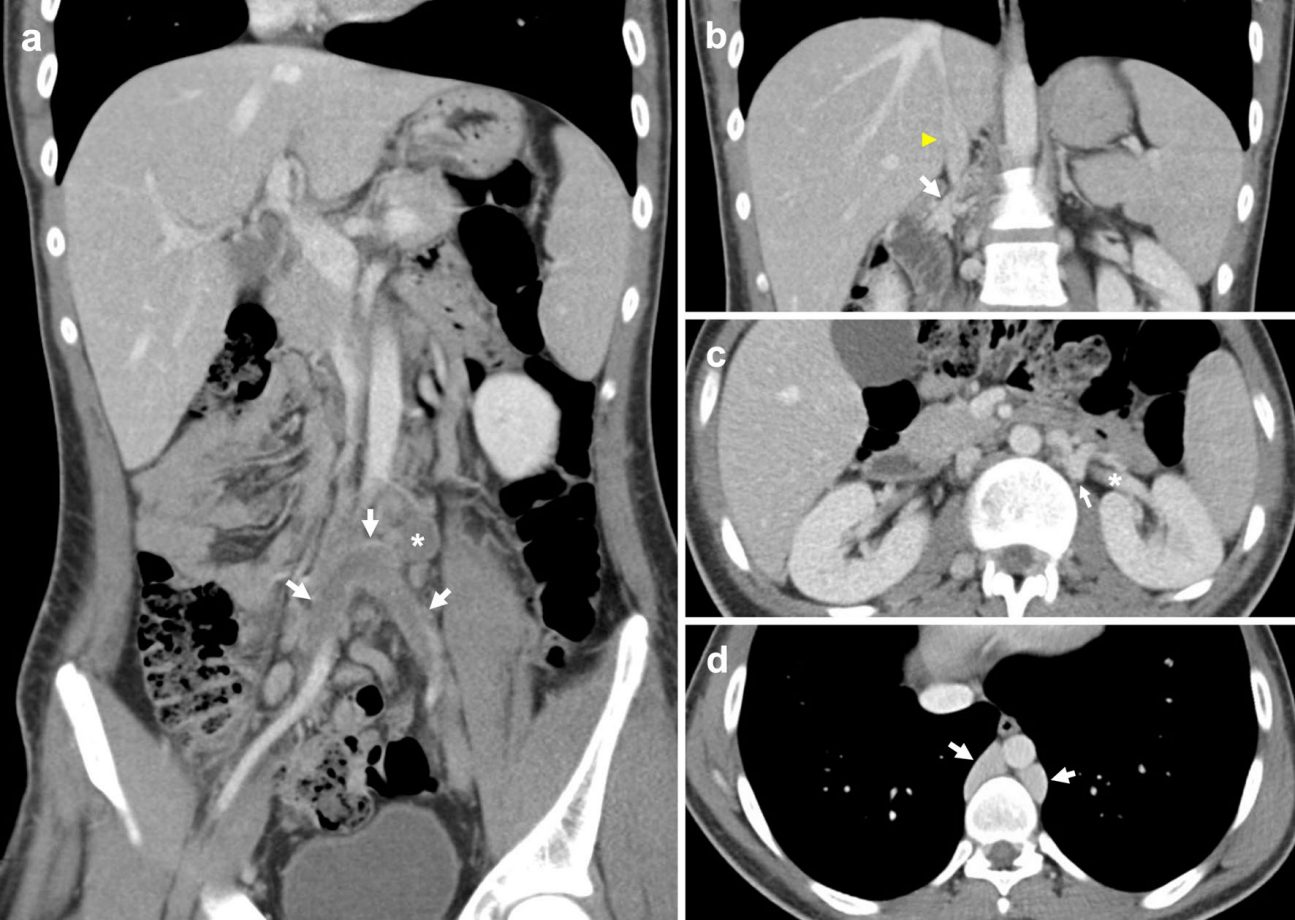
Extensive venous thrombus secondary to interrupted IVC with azygos continuation. A 19-year-old man presented with a right lower limb DVT and underwent contrast-enhanced CT to assess the proximal extent of thrombus. He later developed a right ankle ulcer, likely contributed to by post-thrombotic syndrome. **a** Coronal CT image in the portal venous phase shows occlusive venous thrombus (arrows) in the common iliac veins and an adjacent paravertebral collateral vein (asterisk). Infrarenal and renal segments of the IVC are absent. **b** Coronal CT demonstrates the right renal vein (arrow) draining into a small caliber interrupted inferior vena cava (arrowhead). **c** Axial CT shows a left renal vein (asterisk) that drains into a tortuous left-sided paravertebral collateral vein (arrow). **d** Axial CT image with distended azygos and hemiazygos veins (arrows)

**Fig. 22:**
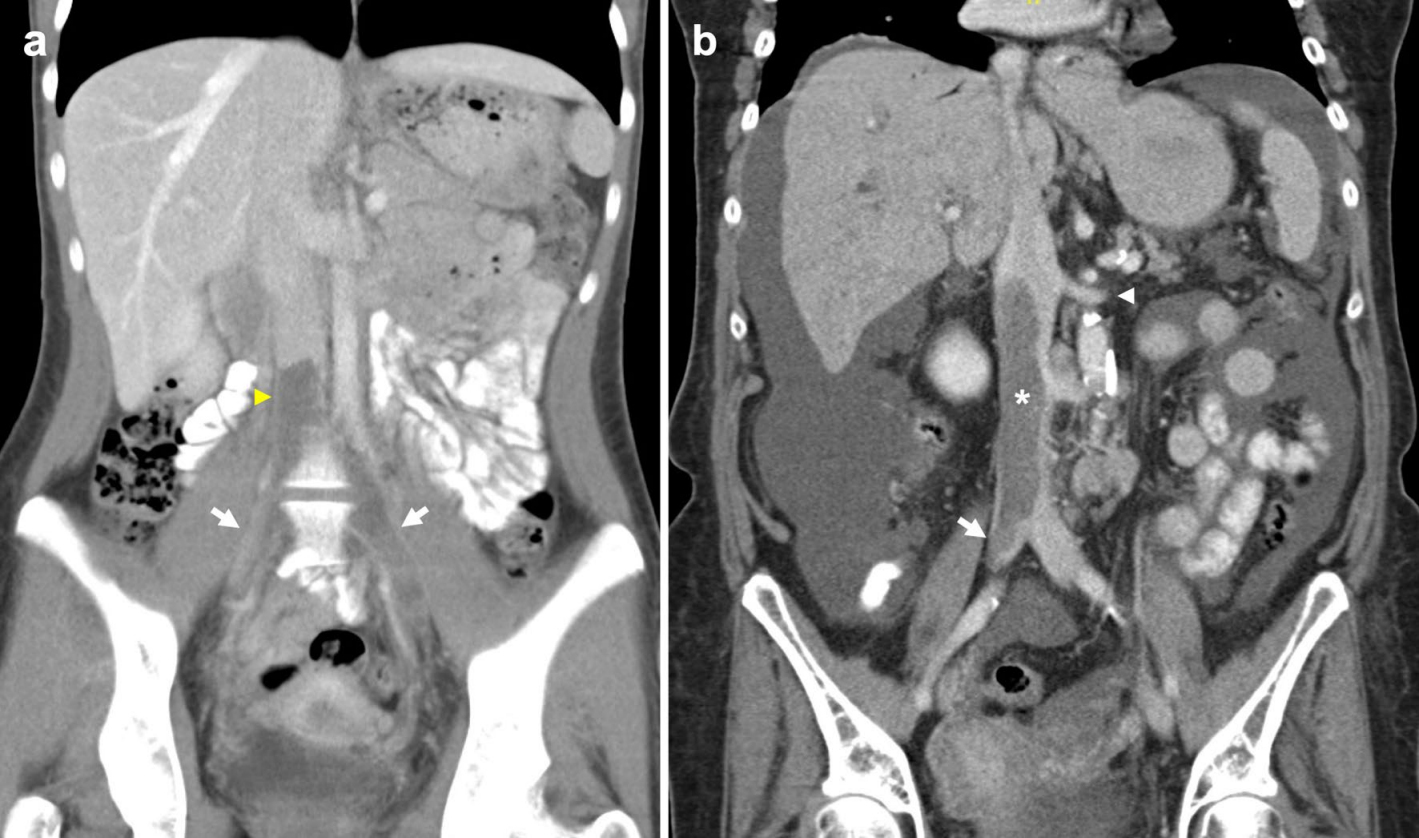
IVC bland thrombosis. **a** A 20-year-old woman presented with an oedematous left leg and lower back pain in the context of smoking, regular oral contraceptive use and previous DVT. Contrast-enhanced coronal CT shows a non-enhancing filling defect in the infrarenal cava (arrowhead) that propagates into bilateral common iliac veins (arrows). Mild intra-abdominal fat stranding is likely secondary to thrombosis-related oedema. Thrombophilia screen was negative, and there was no evidence of malignancy or venous compression on a CT scan of the chest, abdomen and pelvis. **b** A 75-year-old woman presented with a left above knee DVT on a background of cholangiocarcinoma with metastases to the liver and left adnexa. Contrast-enhanced coronal CT demonstrates a partially occlusive non-enhancing inferior vena cava thrombus (asterisk) extending superiorly to the level of the left renal vein (arrowhead), and to the right common iliac vein (arrow) caudally

IVC thrombosis carries significant morbidity and mortality, with a high risk for pulmonary embolism [118]. Commonly reported causes include anomalous venous anatomy, extraluminal or intraluminal obstruction by malignancy, extension of deep vein thrombosis (DVT) and unretrieved IVC filters [119]. 5–16% of young patients presenting with iliofemoral DVT had IVC anomalies, with most cases attributed to IVC interruption/agenesis likely secondary to venous stasis from poor collaterals (Fig. 21) [120–123]. Up to 4% of patients with lower limb DVT experience IVC thrombosis (Fig. 22a) [119]. Common predisposing factors such as malignancy, localised inflammation and coagulopathy cause hypercoagulable states (Fig. 22b). In addition to malignancy-related hypercoagulability, tumour invasion by renal cell carcinoma is an additional independent risk factor for thrombogenesis [57].

Clinical presentation depends on acuity and extent of thrombosis and may vary from incidental detection on imaging, to non-specific back pain preceding symptoms of lower limb venous insufficiency, to cardiopulmonary compromise [124]. If untreated, up to 90% of patients may develop post-thrombotic syndrome, while 45% can develop severe venous claudication [118, 125]. In contrast to tumour thrombus, bland thrombus does not enhance and lacks both luminal expansion and contiguity with the mass [16]. Anticoagulation is the mainstay of treatment, and a subset of patients with acute and subacute thrombus may benefit from catheter-directed thrombolysis with or without angioplasty and stenting [119].

### Pseudothrombus

**Fig. 23:**
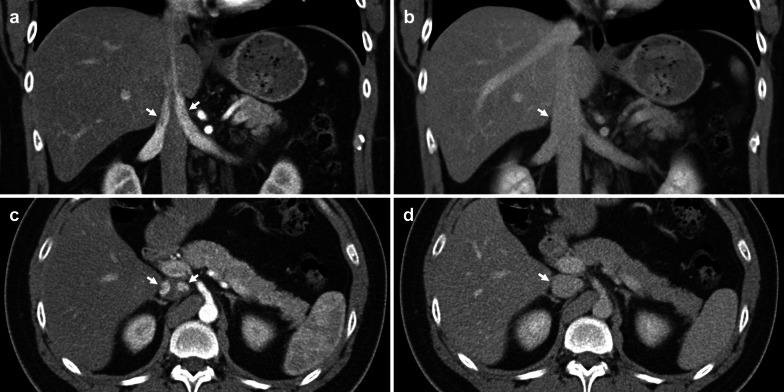
IVC pseudothrombus due to mixing artefact. **a,c** Arterial phase CT demonstrates an apparent filling defect in the IVC attributable to contrast-enhanced blood (arrows) draining from both renal veins and running alongside a column of non-enhanced blood from the lower extremities. **b,d** Coronal and axial CT images of the suprarenal IVC (arrows) show resolution of the filling defect in portal venous phase

Pseudothrombus or ‘mixing artefact’ is a potential imaging pitfall due to heterogeneous opacification of blood mimicking the appearance of a thrombus. This can be seen on both CT and MR imaging, and in the IVC is most often attributed to contrast-enhanced blood from the renal veins flowing alongside a column of unenhanced blood from the lower extremities (Fig. 23) [126]. It can also occur due to retrograde flow of contrast material due to right heart failure [59]. Delayed post-contrast imaging 70–90 s after contrast material injection allows for more uniform opacification of blood, and therefore resolution of the mixing artefact [17].

### Other pathologies

**Fig. 24:**
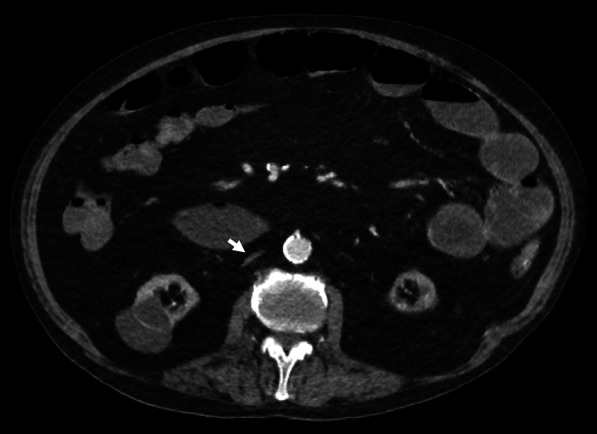
Slit-like IVC from hypovolaemia. Axial-enhanced CT image demonstrates a slit-like IVC or ‘flat cava’ sign (arrow), consistent with the patient’s clinically evident hypovolaemia following surgery

A slit-like or flattened IVC may be defined as an IVC with an anteroposterior diameter less than 9 mm or a transverse-to-anteroposterior diameter ratio greater than 3:1 that is seen at multiple levels (Fig. 24) [127–129]. This is particularly important to recognise in the context of trauma, as it can indicate hypovolaemia or hypotension [17]. Moreover, when not involved in trauma, this sign can predict development of shock, need for aggressive resuscitation and increased mortality [127–130]. However, this should be taken with a grain of salt, as a slit-like IVC may be non-specific outside the context of trauma. A retrospective study of approximately 500 patients without a history of trauma demonstrated that up to two-thirds of patients with a ‘flat cava’ sign, or slit-like IVC, were clinically euvolaemic or normotensive [131].

**Fig. 25:**
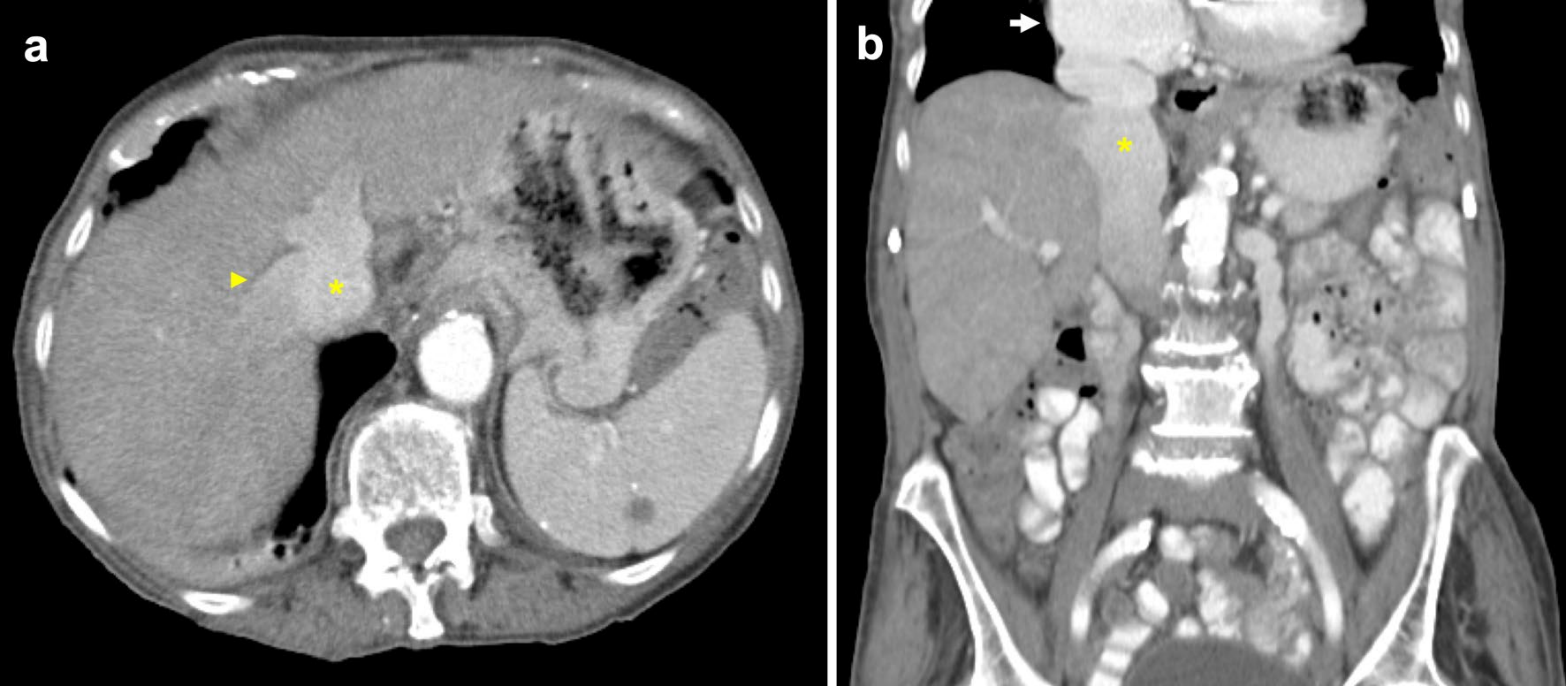
Dilated IVC from heart failure. **a** Axial and (**b**) coronal contrast-enhanced CT images demonstrating dilation of the IVC (asterisk) with hepatic reflux of contrast from the right atrium with early opacification (arrowhead) in a patient with clinically evident heart failure. The cardiomegaly (arrow) is partially imaged

**Fig. 26:**
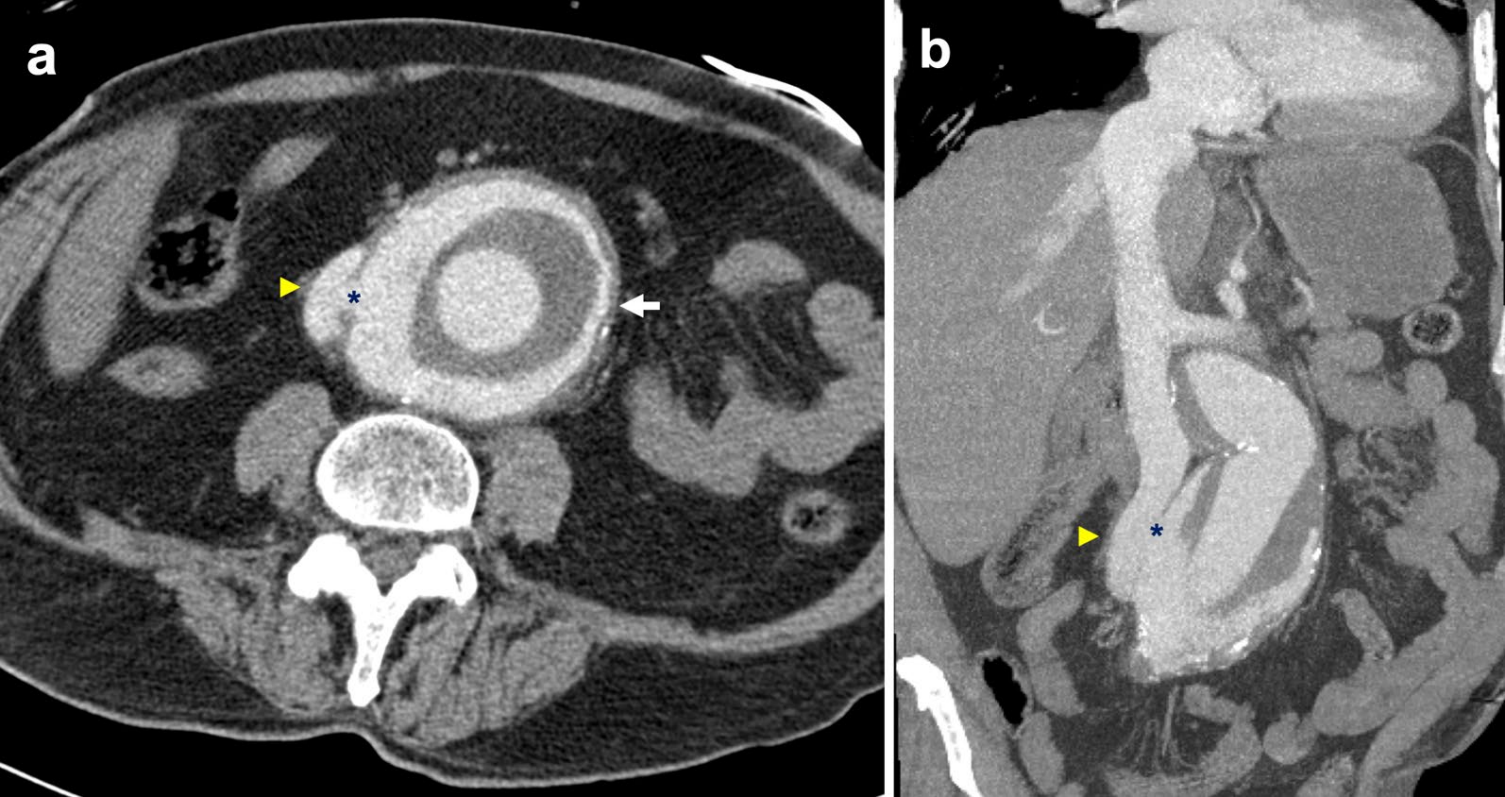
Aortocaval fistula complicating abdominal aortic aneursym. **a** Axial and **b** coronal contrast-enhanced CT images of a fistula (asterisk) between the IVC (arrowhead) and an abdominal aortic aneurysm (arrow), with similar contrast opacification of the aneurysm sac and the IVC in the arterial phase. The abdominal aortic aneurysm contains a combination of contrast opacification and non-enhancing thrombus

Early opacification of the IVC can be seen in a several conditions such as congestive heart failure, aortocaval fistula, arteriovenous shunting or superior vena cava obstruction [16]. Dilation of the IVC and hepatic veins, in addition to early opacification of the IVC and hepatic veins on contrast-enhanced cross-sectional imaging, is characteristic findings of congestive hepatopathy. This can be secondary to passive congestion, most commonly secondary to cardiac disease (Fig. 25) [132]. An aortocaval fistula is an uncommon complication, most often attributed to erosion from an abdominal aortic aneurysm (Fig. 26). Other rarer causes include post-traumatic fistulisation, neoplasm and inflammatory conditions. Timely diagnosis and referral for surgical or endovascular repair are crucial for improved patient outcomes [72].

## Conclusion

The IVC is an important structure that can be affected by a wide range of congenital anomalies and acquired pathologies. It is important for radiologists to be familiar with various appearances of the IVC in benign and malignant pathologies. The recognition of the anatomical variants of the IVC is also important, especially prior to intervention. Imaging also plays a key role in differentiating tumour extension from bland thrombus and in assessing extent of tumour thrombus. Such information is essential for patient care and impacts staging, surgical planning and medical therapy. Evaluation of the IVC should form a fundamental part of a radiologist’s search pattern.

## Data Availability

Not applicable.

## Abbreviations

ACC: Adrenal cortical carcinoma
ADC: Apparent diffusion coefficient
CT: Computed tomography
DVT: Deep vein thrombosis
FDG: Flurodeoxyglucose
HCC: Hepatocellular carcinoma
HU: Hounsfield units
IVC: Inferior vena cava
MRI: Magnetic resonance imaging
NSCLC: Non-small cell lung carcinoma
PET: Positron emission tomography
RCC: Renal cell carcinoma
SPECT: Single-photon emission computed tomography
SVC: Superior vena cava
TNM: Tumour–node–metastasis
VIBE: Volumetric interpolated breath-hold examination
